# Evolution and diversity of floral scent chemistry in the euglossine bee-pollinated orchid genus *Gongora*

**DOI:** 10.1093/aob/mcw072

**Published:** 2016-05-30

**Authors:** Molly C. Hetherington-Rauth, Santiago R. Ramírez

**Affiliations:** ^1^Biology Department, University of Toronto Mississauga, 3359 Mississauga Road North, Mississauga, ON L5L 1C6, Canada; ^2^Department of Evolution and Ecology, University of California, Davis, 1 Shields Avenue, Davis, CA 95616, USA

**Keywords:** Euglossine bees, floral scent, orchid genus *Gongora*, plant–pollinator mutualism, *Euglossa*

## Abstract

**•Background and Aims** Animal-pollinated angiosperms have evolved a variety of signalling mechanisms to attract pollinators. Floral scent is a key component of pollinator attraction, and its chemistry modulates both pollinator behaviour and the formation of plant–pollinator networks. The neotropical orchid genus *Gongora* exhibits specialized pollinator associations with male orchid bees (Euglossini). Male bees visit orchid flowers to collect volatile chemical compounds that they store in hind-leg pouches to use subsequently during courtship display. Hence, *Gongora* floral scent compounds simultaneously serve as signalling molecules and pollinator rewards. Furthermore, because floral scent acts as the predominant reproductive isolating barrier among lineages, it has been hypothesized that chemical traits are highly species specific. A comparative analysis of intra- and inter-specific variation of floral scent chemistry was conducted to investigate the evolutionary patterns across the genus.

**•Methods** Gas chromatography–mass spectrometry (GC-MS) was used to analyse the floral scent of 78 individuals belonging to 28 different species of *Gongora* from two of the three major lineages sampled across the neotropical region. Multidimensional scaling and indicator value analyses were implemented to investigate the patterns of chemical diversity within and among taxonomic groups at various geographic scales. Additionally, pollinator observations were conducted on a sympatric community of *Gongora* orchids exhibiting distinct floral scent phenotypes.

**•Key Results** A total of 83 floral volatiles, mainly terpenes and aromatic compounds, were detected. Many of the identified compounds are common across diverse angiosperm families (e.g. cineole, eugenol, β-ocimene, β-pinene and terpinen-4-ol), while others are relatively rare outside euglossine bee-pollinated orchid lineages. Additionally, 29 volatiles were identified that are known to attract and elicit collection behaviour in male bees. Floral scent traits were less variable within species than between species, and the analysis revealed exceptional levels of cryptic diversity. *Gongora* species were divided into 15 fragrance groups based on shared compounds. Fragrance groups indicate that floral scent variation is not predicted by taxonomic rank or biogeographic region.

**•Conclusions** *Gongora* orchids emit a diverse array of scent molecules that are largely species specific, and closely related taxa exhibit qualitatively and quantitatively divergent chemical profiles. It is shown that within a community, *Gongora* scent chemotypes are correlated with near non-overlapping bee pollinator assemblies. The results lend support to the hypothesis that floral scent traits regulate the architecture of bee pollinator associations. Thus, *Gongora* provides unique opportunities to examine the interplay between floral traits and pollinator specialization in plant–pollinator mutualisms.

## INTRODUCTION

Mutualisms between flowering plants and their insect pollinators have shaped the evolution of floral traits and are thought to contribute significantly to angiosperm diversification (Darwin, 1862; Crepet, 1984; Johnson, 1996; Dodd, *et al.*, 1999; Kay, 2006; Kay and Sargent, 2009; Schiestl and Schlüter, 2009; van der Niet *et al.*, 2014; Breitkopf *et al.*, 2015). Pollinator attraction is often mediated by multimodal signalling mechanisms, including floral morphology, colour and scent (Raguso, 2001; Willmer, 2011). Floral scent is thought to play a central role in mediating pollinator attraction and specificity, especially among highly specialized plant–pollinator interactions (Raguso, 2001; Schiestl and Ayasse, 2002; Mant *et al.*, 2005; Peakall *et al.*, 2010; Willmer, 2011; Xu *et al.*, 2011; Jürgens *et al.*, 2013; Peakall and Whitehead, 2014; van der Niet *et al.*, 2014). Angiosperms emit an exceptionally diverse array of floral scent molecules (Knudsen *et al.*, 1993; Knudsen and Gershenzon, 2006), and many classes of volatile compounds are recurrently associated with specific pollinators, thus suggesting that olfactory sensory mechanisms and behavioural preferences of pollinators have shaped the evolution of floral scent chemistry (Schiestl *et al.*, 2010; Ramírez *et al.*, 2011; Steiger *et al.*, 2011; Schiestl and Dötterl, 2012; Jürgens *et al.*, 2013).

**Fig. 1.**
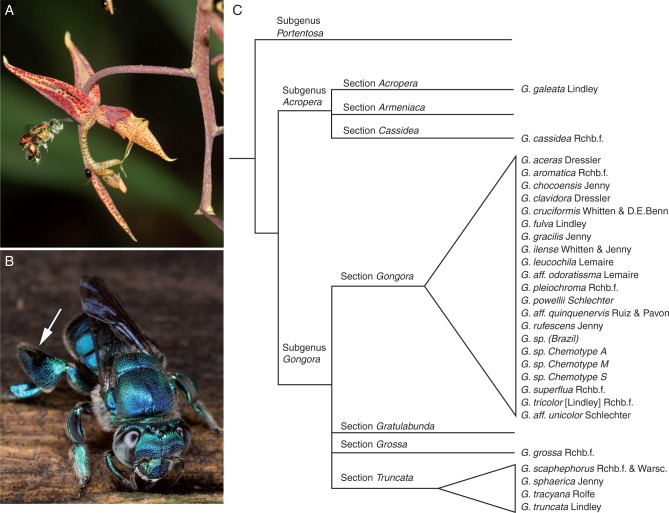
(A) *Gongora* sp. chemotype M (subgenus *Gongora*, section *Gongora*) being visited by *Euglossa dodsoni.* (B) Male euglossine bees collect perfume compounds and store them in hind-leg pockets (arrow) subsequently to expose to females during courtship display (photo: B. Jacobi). (C) Putative phylogenetic relationships of *Gongora* lineages sampled in the present study (as summarized by Hetherington-Rauth and Ramírez, 2015).

The neotropical orchid genus *Gongora* (Orchidaceae: Cymbidieae) exhibits specialized mutualistic associations with scent-collecting male euglossine bees (Fig. 1), in which floral scent volatiles act simultaneously as attractant molecules and insect pollinator rewards (Dodson *et al.*, 1969). Male euglossine bees (Apidae: Euglossini) visit a diverse array of floral sources (e.g. orchids and other angiosperm families) as well as non-floral resources (e.g*.* fungi, rotting vegetation and decaying wood) in order to gather and concoct species-specific perfume blends, which they store in pockets located in the hind tibiae (Eltz *et al.*, 1999). Male bees subsequently expose perfume compounds during courtship to convey information on male quality (fitness) or identity (species) (Eltz *et al.*, 2003). While gathering floral volatiles from orchids, male bees inadvertently remove pollinaria and upon visitation of another flower deposit pollinaria on the stigmatic surface (Allen, 1954; Dressler, 1981; Rodriguez Flores *et al.*, 1995; Hetherington-Rauth and Ramírez, 2015). Thus, reliable floral cues (scents) are needed to ensure species-specific pollination. Because *Gongora* orchids lack additional floral rewards (such as nectar and/or edible pollen) that could potentially attract other pollinators, they rely exclusively on male euglossine bees for sexual reproduction. Between one and five species of eulgossine bee visit a single species of *Gongora* in a given locality (Dressler, 1968*a*; Whitten, 1985; Hentrich, 2004; Hetherington-Rauth and Ramírez, 2015).

Euglossine bees occur throughout the neotropical region, with >230 described species (Ramírez *et al.*, 2002, 2010*b*; Nemesio and Silveira, 2007). Male bees partition chemical niche space by collecting species-specific perfumes (Zimmermann *et al.*, 2009). The chemical phenotype of each species’ perfume is conserved across geography, even among populations inhabiting disparate habitats (Zimmermann *et al.*, 2006; Ramírez, *et al.*, 2010*a*). Therefore, the chemical preferences of male bees probably exert strong selective pressures on the chemistry and phenotypic variation of orchid floral scent (Ramírez *et al.*, 2011). In fact, Dressler (1966) remarked that by placing two varieties of *Gongora* that differed in their perceivable floral scent on either side of a trail, he was able to invent an ‘excellent bee sorter’ (p. 220) with only green bees visiting one variety and only blue bees visiting the other. This demonstrated the potential of floral scent to attract specific pollinator assemblages, and raised the possibility that positive assortative mating could emerge, with variation in floral scent resulting in pre-pollination ethological isolation among orchid populations (Jones, 2001).

The genus *Gongora* contains 60–70 species that are broadly distributed throughout lowland neotropical rain forests (Jenny, 1993). *Gongora* orchids are long-lived perennial epiphytes that produce pendent inflorescences with approx. 10–20 flowers (but some produce up to 50 flowers) that open simultaneously and smell strongest in the morning hours when male bees are most active (Fig. 1A, B) (Hetherington-Rauth and Ramírez, 2015). Flowers typically last a few days before wilting. Although no species-level phylogeny exists for the genus, molecular data support a division of *Gongora* into three subgenera – subgenus *Portentosa*, subgenus *Acropera* and subgenus *Gongora* – and places subgenus *Portentosa* as sister to the clade *Acropera + Gongora* (Whitten *et al.*, 2000, 2014; Hetherington-Rauth and Ramírez, 2015). The subgenus *Gongora* is divided into four sections – section *Gongora*, section *Grossa*, section *Gratulabunda* and section *Truncata* (Fig. 1C). *Gongora* is hypothesized to have experienced a relatively recent diversification within the sub-tribe Stanhopeinae (+Coeliopsidinae) for which euglossine bee pollination is the ancestral state (Whitten *et al.*, 1986; Ramírez *et al.*, 2011; Hetherington-Rauth and Ramírez, 2015). Species diagnoses in *Gongora* are largely based on floral morphology, and although floral scent has a major impact on reproductive isolation, scent traits and/or pollinator associations have not been used to define species. Hence, taxonomic confusion has accumulated, particularly in the subgenus *Gongora* section *Gongora*, where seemingly distinct species exist – as evidenced by the presence of non-overlapping pollinator assemblages – with little morphological differentiation (Dressler, 1966, 1968*a*; Whitten, 1985; Jenny, 1993; Hentrich, 2004; Hetherington-Rauth and Ramírez, 2015).

Although floral scent appears to play a central role in reproductive isolation in *Gongora*, little is known about its chemistry, variation and evolution across the genus. Previous studies on scent chemistry have been restricted to either a few species or populations from a single locality (Hills *et al.*, 1972; Gerlach and Schill, 1991; Kaiser, 1993). Notably, Whitten (1985) and Hentrich (2004) conducted detailed studies of two different species complexes in the genus *Gongora* from central Panama and the central Pacific coast of Costa Rica, respectively. Each study identified putative cryptic species through the combination of chemical analyses of floral scent and pollinator observations, and both clearly demonstrated that (1) otherwise indistinguishable individuals produce qualitatively distinct scent phenotypes and (2) floral scent phenotypes correlate with non-overlapping assemblages of bee pollinators. These studies highlight that phenotypic variation in floral scent can influence the formation of specialized plant–pollinator networks, which in turn can lead to the formation of reproductive barriers.

We conducted a comparative analysis of floral scent chemistry using approx. 28 species of *Gongora* from two of the three subgenera with taxa sampled from a broad geographic distribution. In particular, we asked the following questions. (1) What floral volatiles are present in the floral scent? (2) Do species produce species-specific scent bouquets as hypothesized by the existence of strong ethological reproductive isolation and, if so, can floral scent traits inform species delimitation? (3) Are scent chemotypes correlated with the identity of bee pollinator assemblages? (4) Is floral scent taxonomically conserved among subgenera and sections? (5) How are floral scent phenotypes distributed across geography with emphasis on sympatric lineages?

## MATERIALS AND METHODS

### Plant material

*Gongora* orchids were cultivated in a greenhouse facility at the University of California, Davis. In addition, several species used in this study were maintained in outdoor nurseries near Virterbo (Colombia) and La Gamba Tropical Field Station near Golfito (Costa Rica). Plants from La Gamba were collected from the surrounding Esquinas Rainforest on the southern Pacific peninsula of Costa Rica. Plants were identified to species based on floral characters. Individuals that could not be unambiguously identified to species were given a species *affinis* (aff.) name. No species names are available for La Gamba *Gongora* and thus these are referred based on their scent profiles: chemotype A, chemotype S and chemotype M. We only used individuals with known and/or inferred collection localities (Supplementary Data Table S1).

### Collection of floral volatiles

We sampled floral scent using a static headspace method (Williams and Whitten, 1983; Tholl *et al.*, 2006) from plants kept in the greenhouse or in the field. Sampling was conducted between 0800 and 1300 h on the first, second or third day of anthesis, which corresponds to the time when euglossine bees are most active and floral scent production peaks (Whitten, 1985; Hills, 1989; Hills and Williams, 1990). We bagged one inflorescence with nylon oven bags (Reynolds Kitchens, Richmond, VA, USA) closed at the top with metal wire (Stewart-Jones and Poppy, 2006). Inflorescences were bagged for 30 min. Subsequently, we connected scent traps to an electrical vacuum pump (Parker, Cleveland, OH, USA) via Tygon tubing (ID 3·3 mm) and continuously extracted air from the bag through a small slit. Single-use scent traps were constructed using clear glass tubing (2·4 mm ID, 3·5 cm length) plugged at both ends with glass wool and filled with 20 mg of bulk carbide (charcoal) and 20 mg of Tenax GC (SUPELCO, Bellefonte, PA, USA; mesh size 60/80) (Williams and Whitten, 1983; Whitten, 1985; Raguso and Pellmyr, 1998). Scent traps were conditioned by passing 5 mL of hexane. Air was pulled from the headspace through the scent trap at 2·5 L min^−1^ for 2 h. Scent traps were eluted with 200 μL of hexane into conical inserts (Agilent Technologies, Santa Clara, CA, USA) held in 2 mL auto-sampler vials (Agilent Technologies). Auto-sampler vials were capped with screw cap PTFE/silicon lids (Agilent Technologies) and stored at –20 °C until analysed. Control samples were collected simultaneously in the same manner from oven bags filled with ambient air. Samples were acquired between January 2014 and April 2015. Whenever possible, we sampled three unique inflorescences per plant.

### Gas chromatography–mass spectrometry (GC-MS) analysis

We analysed samples with an Agilent 7890B GC fitted with a 30 m × 0·25 mm × 0·25 μm HP-5 Ultra Inert column coupled to an Agilent 5977A mass spectrometer (Agilent Technologies). Using an auto-sampler, we injected 1 μL into the gas chromatograph at a 5:1 split ratio. The split ratio was adjusted for some samples to increase detection thresholds (see Table S1). Oven temperature was held at 60 °C for 3 min and then increased by 10 °C min^−1^ until it reached 300 °C; then the oven temperature was kept at 315 °C for 1 min. Both injector and transfer line temperatures were kept constant at 250 °C. Helium served as the carrier gas with a constant flow rate set to 1·2 mL min^−1^. Electron impact mass spectra were obtained by scanning between 30 and 550 *m/z*. GC-MS data were processed using MassHunter GC/MS Acquisition software vB.07.00 (Agilent) and MSD ChemStation Enhanced Data Analysis Software vF.01.00 (Agilent).

### Compound characterization

We tentatively identified individual compounds using the NIST05 mass spectral database and the NIST MS Search software v2.0. We confirmed compound identification by comparing relative retention times of authentic chemical standards run under the same conditions. In addition, we compared the calculated Kovats Retention Indexes, estimated with a series of alkane standards (C7–C30), with that of published data (Adams, 2007). For compounds that we were unable to identify unambiguously, we list their EI mass spectrum ions. We removed putative contaminant compounds when present with comparable peak areas (within a factor of 10×) in both the control and the sample. We determined total ion abundances by integrating peaks in the MSD ChemStation software using the RTE integrator. Only peaks with an area ≥3 % of the largest peak were included in downstream analyses. When compound co-elution occurred, de-convolution was performed using AMDIS software v2.64. The total peak area was partitioned among co-eluting peaks based on the relative areas of two to three diagnostic ions. Compounds contributing less than one-tenth to the total peak area were discarded.

### Statistical analysis

We implemented multivariate statistical approaches to investigate the variation of chemical profiles among individuals and taxa. Briefly, we normalized raw chromatogram peak areas by calculating the contribution of each compound relative to the total area. We averaged the relative proportion of each compound across samples of the same individual. In addition, we created a binary matrix (presence/absence) in which all compounds are equally weighted. We calculated pairwise distance among individuals for both relative proportions and binary values using the Bray–Curtis dissimilarity metric in the package ‘ecodist’ v1.2.9. The Bray–Curtis dissimilarity metric is unaffected by ‘double zeros’ and only considers compounds that are jointly shared between individuals (Beals, 1984; Zimmermann *et al.*, 2009; Legendre and Legendre, 2012). The dissimilarity matrices were used to conduct non-metric multidimensional scaling (nMDS) analysis. This method visually represents similarity amongst individuals in pre-specified reduced space dimensions, using a non-eigenvector approach that allows for a flexible choice of distance metrics (e.g. Bray–Curtis) (Jones, 2001; Zimmermann *et al.*, 2009). We constructed two-dimensional plots using two different algorithms: the ‘metaMDS’ algorithm in ‘vegan’ v2.2-1 and the ‘nmds’ algorithm in ‘ecodist’ v1.2.9. Each algorithm produced similar plots but differed in that the ‘vegan’ algorithm tended to minimize variation relative to the ‘ecodist’ algorithm. We used 20 random starting configurations. The resulting inter-point distances attempt to maximize the rank-ordered chemical distances among individuals (Clarke and Warwick, 2001). Individuals clustering together share a similar floral scent composition. The stress value (ranging from 0 to 1) associated with the nMDS plot reflects how well the algorithm preserved the rank-ordered distance measures. Stress values <0·2 are desirable when calculated using the Kruskal equation (Kruskal, 1964; Clarke and Warwick, 2001; Legendre and Legendre, 2012). The ‘vegan’ package calculates the stress value according to the Kruskal equation, whereas the ‘ecodist’ package uses a variation of the Kruskal equation. Stress values calculated with ‘vegan’ are consistently smaller than those calculated with ‘ecodist’. We report both.

To identify the components of floral scent that contribute to the observed patterns of variation in chemical space, and to define discrete fragrance groups, we performed an INDicator VALue (INDVAL) analysis (Dufrene and Legendre, 1997; El-Sayed, 2014). INDVAL analysis begins with a set of natural groupings (defined *a priori*) based upon the chemical similarity between individuals. For each group, an indicator value is calculated for every compound. The indicator value accounts for both compound specificity (the percentage of individuals within the group that produces that compound) and compound fidelity (the percentage of groups in which that compound is present). The indicator value is maximum (=1) when a compound is present in only a single group and each individual within that group produces that compound. We computed indicator values for each compound for a range of *k*-values (1–20), indicating the number of group clusters. Clusters were determined manually from a clustering dendrogram constructed using the dissimilarity matrix that accounted for the relative proportions of compounds and an UPGMA (unweighted pair-group method using arithmetic averages) agglomerative clustering method as implemented in the R software package ‘cluster’ v1.15.2. The significance of the indicator value was evaluated using a random permutation method. We used the *k-*value for which the summation of the significant indicator values (*P* < 0·05) was maximum; this provides a natural number of clusters or fragrance groups present in the data. Compounds with significant indicator values (*P* < 0·05) are called indicator compounds and can be considered as diagnostic of each fragrance group. Thus, INDVAL analysis provides a concise summary of the compounds that distinguish species and species groups. INDVAL analysis was carried out using the R package ‘labdsv’ v1.4–1.

To test whether the observed differences in floral scent composition could be explained by *a priori* defined groups (subgenera, species, coloration and locality), we implemented ANOSIM one-way permutation tests with 999 random permutations using the software package ‘vegan’ v2.2-1 (Clarke, 1993; Clarke and Warwick, 2001). The test statistic, *R*, is calculated using the rank similarities as calculated from the chemical dissimilarity matrix and is a measure of the difference in the rank similarities within and between groups (Clarke, 1993). The null hypothesis is that there are no differences between *a priori* groups*. R*-values close to 1 indicate greater between-group dissimilarity than within-group dissimilarity (the null hypothesis can be rejected), whereas an *R* close to 0 indicates no difference between within-group and among-group dissimilarities (null cannot be rejected).

### Euglossine bee flower visitors

Between 2013 and 2015, we documented the euglossine bee species that visited La Gamba *Gongora.* Plants were collected from the Esquinas Rainforest near La Gamba Tropical Field Station. Plants in bloom were taken to the field in the morning after headspace samples were taken. Between 0900 and 1200 h we recorded visitors (bees that landed and actively collected floral scent). All bee visitors were captured and their behaviour recorded. We visualized pollinator networks using the R package ‘bipartite’ v2.05.

### Biogeographic patterns of floral scent

We examined the distribution of floral scent in the genus *Gongora* across geography. We assigned individuals in our dataset to nine pre-specified biogeographic regions (Gentry, 1982). These regions show high levels of recurrent endemism (Gentry, 1982; Morrone, 2006). For individuals whose collection locality was not precise, we used the species range distribution and type localities as proxies (Jenny, 1993). We examined the distribution of INDVAL fragrance groups across biogeographic regions.

## RESULTS

### Sampling

We sampled floral scent from 88 individuals representing 28 taxonomically described species from two subgenera and three of the four sections of the subgenus *Gongora* (Fig. 1C; Supplementary Data Table S2). For 12 individuals, we used the splitless injection mode to better resolve chromatograms (see Table S1). These individuals did not cluster together in our analysis, suggesting that the alternative GC injection method did not influence our results. Splitless injection was conducted for individuals of *G. chocoensis*, *G. leucochila*, *G. gracilis*, *G. pleiochroma* and *G.* aff. *unicolor.* In addition, four species (nine individuals) exhibited noticeable smell but consistently produced chromatograms without peaks (*G. leuchochila*, *G. chocoensis*, *G. superflua* and *G.* aff. *unicolor*). Scent composition of all individuals is given in Supplementary Data Tables S3A (averaged across replicates) and B (averaged across individuals).

We examined within-individual variation across replicate samples using a clustering dendrogram based on the Bray–Curtis dissimilarity metric (Supplementary Data Fig. S9). With few exceptions, replicates of the same individual consistently clustered tightly together, indicating that both individual-level variation and instrument error were negligible relative to the variation observed between taxa. Moreover, our results remained unchanged whether we included or discarded the three replicate samples that failed to cluster tightly among replicates. Additionally, replicates did not cluster by day after anthesis (either 1, 2 or 3), indicating that the overall scent chemistry remains constant during the lifetime of the inflorescence.

### Chemical diversity of floral volatiles

We detected a total of 83 floral volatile compounds, 64 of which we identified as known molecules based on mass spectra and comparison with authentic standards (Table S3A, B). Emitted compounds fell into three distinct chemical classes (Knudsen *et al.*, 1993; Dudareva and Pichersky, 2006; Knudsen and Gershenzon, 2006; El-Sayed, 2014) and included 31 aromatic compounds, 31 terpenes (divided between 15 monoterpenes and 16 sesquiterpenes) and one nitrogeneous compound. Many of the identified compounds are commonly emitted by diverse angiosperm families (e.g. 1,8-cineole, eugenol, β-ocimene, β-pinene and terpinen-4-ol) (Knudsen *et al.*, 1993; Dudareva and Pichersky, 2006; Knudsen and Gershenzon, 2006; El-Sayed, 2014), whereas other compounds are relatively rare outside of euglossine bee-pollinated orchid lineages (e.g. dimethoxybenzene, methyl benzoate, *cis*-methyl *p*-methoxycinnamate, ipsdineol, chavicol, anethole, isoeugenol, 5-indanol and *trans*-geranyl acetone) (Gerlach and Schill, 1991).

**Fig. 2.**
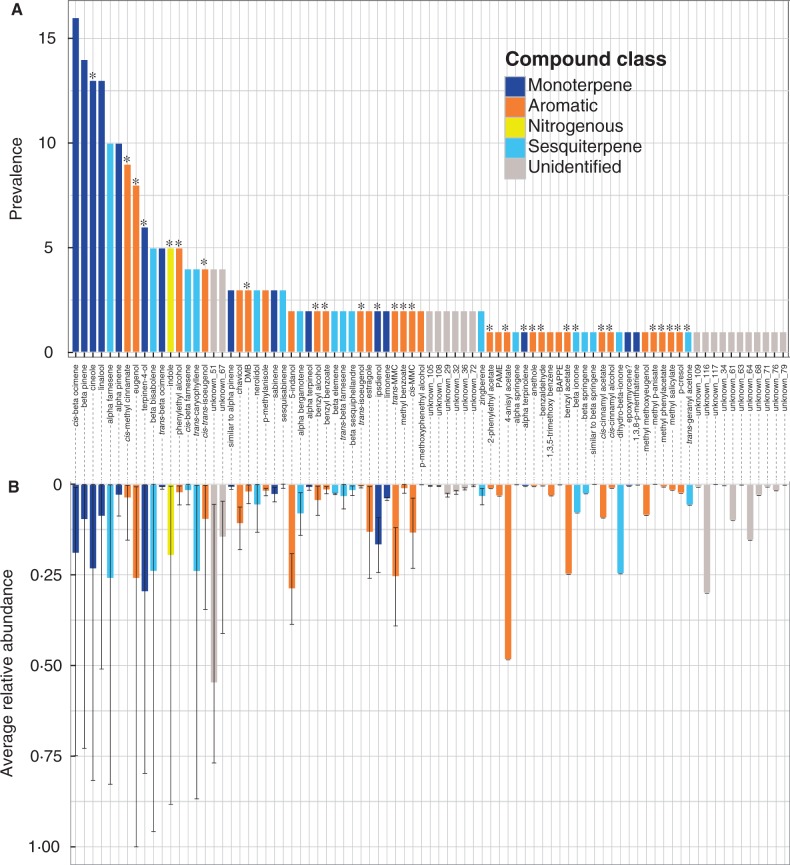
Floral volatile diversity across sampled species. (A) Bar graph depicting compound prevalence. Compound prevalence refers to the number of sampled species that produced a given compound. Compounds are arranged along the *x*-axis in order of decreasing prevalence. Colours represent the chemical class of the compound, including unidentified compounds. Asterisks above the bars indicate floral volatiles that are known to attract and elicit collection behaviour in male euglossine bees. (B) Bar graph indicating the average relative abundance for each floral volatile. Lines laid on top of the bars indicate the maximum and minimum relative abundance values. Compounds along the *x*-axis are arranged in the same order as in (A). Colours correspond to the colours used in (A) Compound abbreviations are as follows: DMB, dimethoxybenzene; MMC, methyl methoxycinnamate; PAME, phenylacetic methyl ester; BAPEEE, benzonic acid *p*-ethoxy ethylester.

The total number of floral volatiles per individual ranged from one to 17, with an average of 5·6 compounds (s.d. = 3·0 and median = 5·0). Few compounds were shared across multiple species, with only 12 compounds identified in five or more species (Fig. 2A). The compounds *cis*-β-ocimene, β-pinene, linalool, 1,8-cineole, α-farnesene and α-pinene, all of which are terpenes, were among the most prevalent compounds, found in 16, 14, 13, ten and ten species, respectively. However the relative contribution of these compounds to the total floral scent bouquet of an individual was highly variable across species (Fig. 2B). For example, the compound *cis*-β-ocimene was identified in 16 species but ranged from 74·9 % in *Gongora cruciformis* to 0·9 % in *G. aceras.* Most compounds (58) were produced by only one or two species, usually in relatively low concentrations, and were mainly either aromatic compounds or unidentified molecules. We found a weak relationship between the prevalence of a compound and its average relative abundance (Pearson’s *R* = 0·295, *P* = 0·006). In general, the relative chemical composition of floral scent among taxa was highly uneven, with a single compound contributing on average 68·7 % (s.d. = 19·6 %, median = 72·0 %) to the total floral scent bouquet. Notably, the phylogenetically restricted compounds, *cis*-methyl *p*-methoxycinnamate, 5-indanol and unidentified compound 51 [probably a monoterpene derivative (F. Zakharov pers. comm.)], were emitted in high relative abundances and contributed disproportionately to the total floral scent bouquet. Additionally, half of all compounds contributed <2·9 % to the total floral scent bouquet and three-quarters of all compounds contributed <10·5 % to the total floral scent bouquet (Fig. 2B).

We compiled information on the attractiveness of each compound from the literature, including field behavioural assays (Dodson *et al.*, 1969; Williams and Dodson, 1972; Williams and Whitten, 1983; Ackerman, 1989) and personal observations. We considered a compound attractive when it was reported that bees were attracted and displayed collection behaviour at the compound exposed in pure form. We identified 29 compounds as attractive (Fig. 2B). There was no apparent association between either the prevalence or the relative abundance of a compound and whether the compound is attractive to male bees.

### Fragrance groups

Our INDVAL analysis revealed broad patterns of chemical diversity across the genus (Table 1). The summation of indicator values was maximal with *k* = 15 clusters. Each of the 15 fragrance groups was distinguished by a set of 1–5 indicator compounds. Generally, indicator compounds exhibited high relative abundance among individuals within the group. An indicator value of 1 (maximum) suggests that the compound is present in only a single fragrance group and each individual within that group produces the compound. An indicator value <1 suggests that (*a*) the compound is not present in all individuals of the fragrance group; (*b*) the compound may be present in individuals not included in the fragrance group; or (*c*) a combination of both. For example, the compounds benzyl acetate and 4-anisyl acetate of fragrance group #15 each have an indicator value of 1. Both compounds are found exclusively in all *Gongora grossa* individuals (*n* = 5). Conversely, the compound *cis-*β-ocimene of fragrance group #1 has an indicator value of 0·63, which is explained by the presence of this compound in taxa outside of fragrance group #1. In fact, *cis*-β-ocimene was the most prevalent compound across all samples. However, inspection of the taxa in fragrance group #1 (*G. aromatica*, *G. cruciformis*, *G. pleiochroma* and *G. powellii*) shows that the floral scent of each species is consistently characterized by a high relative abundance of *cis*-β-ocimene whereas individuals outside fragrance group # 1 produce relatively low abundances of this compound. Similarly, chavicol (fragrance group #7; indicator value = 0·69), 1,8-cineole (fragrance group #13; indicator value = 0·59) and (–) α-pinene (fragrance group #13; indicator value = 0·52) deviated from 1. This observation corroborates that highly prevalent compounds vary considerably in their relative contribution to the floral scent among taxa (Fig. 2A, B). In contrast, only individuals within fragrance group #4 (*G.* sp. chemotype A) produced anethole (indicator value = 0·5). However, this compound was produced by only three of the six individuals in this group. We found a similar pattern for 2-phenylehtyl acetate (fragrance group #7; indicator value = 0·67), 1,3,5-trimethoxy benzene (fragrance group #9; indicator value = 0·5) and unidentified compound #79 (fragrance group #11; indicator value = 0·67).

**Table 1.** Results from the INDVAL analysis indicating the 15 fragrance groups and the associated indicator compound(s)

Fragrance group	Indicator value	*P*-value	Frequency	Indicator compound	Taxa in fragrance group	No. of individuals
1	0·63	0·001	38	*cis-*β-Ocimene	*G. aromatica*	1
					*G. cruciformis*	1
					*G. pleiochroma*	5
					*G. powellii*	7
2	0·77	0·001	34	Linalool	*G. pleiochroma*	9
3	0·99	0·001	4	β-Sesquiphellandrene	*G. pleiochroma*	2
	0·91	0·001	11	β-Bisabolene	*G. superflua*	1
	0·71	0·032	6	*trans*-β-Farnesene		
4	0·99	0·001	9	Estragole	*G.* sp. chemotype A	6
	0·92	0·001	12	MMC	
	0·87	0·001	12	MMC	
	0·50	0·04	3	Anethole		
5	0·98	0·001	6	Unidentified compound #51	*G.* sp. chemotype S	2
					*G. aceras*	2
					*G.* afff. *odoratissma*	1
6	0·98	0·001	10	Terpinen-4-ol	*G.* sp. chemotype M	4
	0·86	0·001	6	Sabiene	*G.* aff. *quinquenervis*	1
	0·86	0·001	6	Limonene	*G. tracyana*	2
7	1·00	0·001	3	5-Indanol	*G.* aff. *unicolor*	2
	1·00	0·001	3	Unidentified compound #105	*G. leuchochila*	1
	0·96	0·002	5	*trans*-Isoeugenol		
	0·69	0·002	9	Chavicol		
	0·67	0·042	2	2-Phenylethyl acetate		
8	0·89	0·001	11	Eugneol	*G.* aff. *unicolor*	2
					*G. clavidora*	1
					*G.* sp. (Brazil)	1
9	1·00	0·001	2	Unidentified compound #116	*G.* aff. *unicolor*	2
	0·5	0·047	1	1,3,5-Trimethtoxy benzene?		
10	0·76	0·0001	18	α-Farnesene	*G.* aff. *quinquenervis*	1
					*G. cassidea*	2
					*G. galeata*	3
					*G. truncata*	1
11	1·00	0·001	3	Ipsdienol	*G. fulva*	2
	0·82	0·001	20	β-Pinene	*G. tricolor*	1
	0·67	0·027	2	Unidentified compound #79		
	0·67	0·032	2	Epoxymyrecene?		
12	0·95	0·012	5	*trans-*Caryophyllene	*G. chocoensis*	1
	0·89	0·008	5	Nerolidol		
13	0·59	0·002	25	1,8-Cineole	*G. ilense*	1
	0·52	0·007	14	(–) α-Pinene	*G. rufescenes*	1
					*G. scaphephorus*	1
					*G. sphaerica*	2
14	0·96	0·001	9	Indole	*G. gracilis*	2
					*G.* aff. *gracilis* (Venezuela)	1
15	1·00	0·001	5	Benzyl acetate	*G. grossa*	5
	1·00	0·001	5	4-Anisyl acetate		
	0·89	0·001	7	DMB		
	0·8	0·010	4	*cis-*Cinnamyl acetate		

The indicator value and *P*-value are given for each compound. The frequency refers to the total number of individuals in which the indicator compound was detected across all 79 sampled individuals. In addition, the corresponding number of individuals within each taxon is indicated.

Floral scent did not appear to be conserved across taxonomic rank. The number of species within each fragrance group ranged from one to four, and the distribution of fragrance groups was heterogeneous across subgenera and sections. Several species formed their own fragrance groups (*G.* sp. chemotype A, *G. chocoeneis*, *G. gracilis* and *G. grossa*)*.* However, more than one species was present in nine of the 15 fragrance groups, suggesting that heterospecific individuals often exhibit similar scent phenotypes due to the presence of shared compounds. Some individuals of the same taxa were split across more than one fragrance group, potentially revealing cryptic taxa. For example, *G. pleiochroma* was divided among fragrance groups #1, #2 and #3, *G.* aff. *unicolor* was divided among fragrance groups #7, #8 and #9, and *G.* aff. *quinquenervis* was divided among fragrance groups #6 and #10. Furthermore the La Gamba *Gonogra* grouped according to their respective chemotypes across fragrance groups #4, #5 and #6.

### Intra- and inter-specific variation in floral scent

**Fig. 3.**
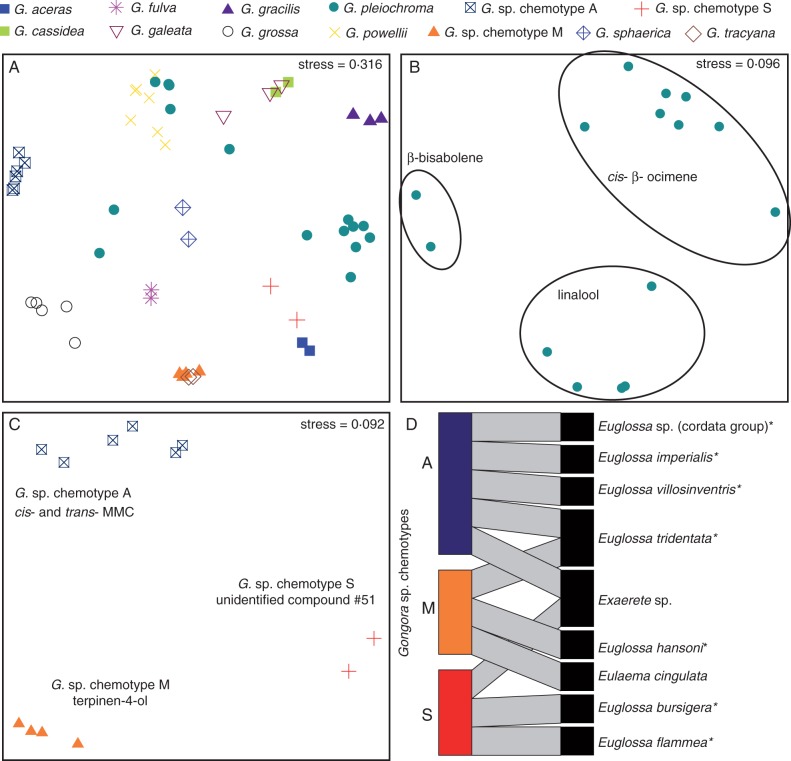
Non-metric multidemsional scaling (nMDS) of floral scent projected in two-dimensional space using the Bray–Curtis dissimilarity metric implemented using the R software package ‘ecodist’. The stress value reported was calculated using the nMDS algorithm in ‘ecodist’. (A) Plotted results of nMDS analysis using a sub-set of species (*n* = 12). Coloured symbols represent separate species. (B) Plotted results of nMDS analysis of *Gongora pleiochroma* individuals. Ellipses correspond to the three chemical phenotypes that differ in the relative contribution of *cis-*β-ocimene, linalool and β-bisabolene to the overall floral scent bouquet. (C) Plotted results of nMDS analysis of La Gamba *Gongora*. Groupings correspond to three identified chemotypes – A, M and S – characterized by *cis-* and *trans*-methyl methoxycinnamate (MMC), terpinen-4-ol and unidentified compound #51, respectively. (D) Bipartite network showing bee–orchid associations between the three chemotypes of La Gamba *Gongora* and nine species of euglossine bees observed actively collecting floral scent from the flowers. Bee species marked with an asterisk indicate species observed carrying *Gongora* pollinaria at chemical baits (S. R. Ramírez unpubl. res.).

We implemented nMDS analyses to investigate the extent of intra- and inter-species variation in floral scent chemistry. In Fig. 3A we plotted 12 species that were represented by at least two individuals (stress in ‘ecodist’ = 0·316, stress in ‘vegan’ = 0·114); however, we excluded *G.* aff. *unicolor* and *G.* aff. *quinquenervis* because of the uncertainty associated with their species identity. We observed relatively less variation within species than between species (ANOSIM: *R* = 0·7223, *P* < 0·001); however, an exception was *G. pleiochroma*, which displayed considerable variation. In some cases, individuals belonging to unrelated species (e.g. *G. tracyana* and *G.* sp. chemotype M) overlapped considerably with one another, suggesting convergence in floral scent chemistry, as was also revealed by the INDVAL analysis.

Our *Gongora* collection contained 17 individuals belonging to *Gongora pleiochroma* collected from a broad geographic range in western Ecuador and Peru, thus providing a unique opportunity to examine intra-specific variation. Our analysis revealed substantial quantitative, but not qualitative, intra-specific variation in scent composition*.* Individuals of *G. pleiochroma* varied slightly in overall flower size and exhibited some morphological differences including the triangular shape of the hypochile, length of the horns and the angle between the lateral and lower sepals. Flower colour varied substantially, ranging from yellow with brown speckles to uniform red/brown with some variation therein (Supplementary Data Figs S5 and S6). Our nMDS analysis revealed three distinct chemical phenotypes (Fig. 3B) associated with *G. pleiohcorma* (stress in ‘ecodist’ = 0·096; stress in ‘vegan’ = 0·0), which corroborates the INDVAL results in which species were split among three fragrance groups (ANOSIM: *R* = 1, *P* < 0·001, *n* = 3 groups based on clusters from INDVAL analysis). The three clusters were not associated with flower colour (ANOSIM: *R* = –0·044, *P* = 0·593, *n* = 2 groups either yellow with brown speckles or uniform red/brown) or with collection locality (ANOSIM: *R* = – 0·054, *P* = 0·601, *n* = 2 groups by biogeographic region). Close inspection of each cluster revealed differences in the relative contribution of *cis*-β-ocimene, linalool and β-biasbolene to the overall floral scent bouquet. When we transformed data into binary (presence/absence) characters, the three clusters reduced to one in chemical space (plot not shown; stress in ‘ecodist’ = 0·250; stress in ‘vegan’ = 0·099; ANOSIM: *R* = 0·221, *P* = 0·053, *n* = 3 groups based on clusters from INDVAL analysis).

Our analysis of La Gamba *Gongora* revealed the presence of three distinct chemical phenotypes that occur in sympatry (Fig. 3C). These individuals were morphologically similar but exhibited exceptional colour variation (Supplementary Data Fig. S7). Individuals were either uniform pale yellow, uniform blush red or yellow to light yellow with red/brown speckles. The red/brown speckles in some cases extended along the entire labellum and in others were restricted to either the hypochile or the epichile. Chemical traits revealed three distinct groups (chemotypes A, S and M; Fig. 3C; stress in ‘ecodist’ = 0·092; stress in ‘vegan’ = 0·0). An ANOSIM test using chemotype for group assignment confirmed the presence of three distinct clusters (*R* = 1·00, *P* < 0·001). The three chemotypes were not clearly associated with colour or morphology. With the exception of 1,8-cineole, which was observed in only low relative abundance in chemotypes S and M, the floral scent of each group was completely distinct and exhibited no overlap of compounds. Our INDVAL analysis indicated that chemotype A is characterized by estragole, *cis*- and *trans-*methyl *p*-methoxycinnamate and anethole (corresponding to fragrance group #4); chemotype S is characterized by unidentified compound #51 (corresponding to fragrance group #5); and floral scent of chemotype M is characterized by terpinen-4-ol, sabiene and limonene (corresponding to fragrance group #6).

### Bee visitor network

We conducted pollinator observations of the three La Gamba chemotypes using ten unique individuals. We accumulated approx. 30 h of field observations, and managed to record a total of 27 bee visitors belonging to nine different species of euglossine bees out of approx. 30 species known from the region. Furthermore, with the exception of *Euleama cingulata* and *Exaerete* sp., each of the observed visiting bee species have previously been observed and collected carrying *Gongora* pollinaria at chemical baits (S. R. Ramírez, unpubl. data), suggesting that the observed visiting bee species probably act as true pollinators. We constructed a bipartite bee–orchid network that revealed a predominant pattern of near non-overlapping bee assemblages associated with each of the three chemotypes present in La Gamba (Fig. 3D). We observed four, three and three species of euglossine bees visiting chemotypes A, M and S, respectively. *Exaerete* sp. and *Euglossa tridentata* visited more than one chemotype; however, *Exaerete* sp. is unlikely to be a true pollinator due to its larger size. Our network reflects a highly specialized plant–pollinator association, and no additional insects were observed as visitors.

### Biogeographic patterns of floral scent

We identified eight biogeographic regions within the geographic range of *Gongora* based on high endemism units (Gentry, 1982). These included (1) Mesoamerica, (2) Ithsmian, (3) Northern Andes, (4) Southern Andes, (5) Northern Venezuela–Colombia, (6) Amazonia, (7) Cerrado and (8) Coastal Brazil. We assigned individuals to fragrance groups and plotted their distribution across these regions (Table 2). Five species were assigned to Meosamerica, eight to the Ithsmian region, 12 to the northern Andes and three to the southern Andes. We were unable to assign *G. gracilis* collected from Venezuela and *G.* sp. collected from Brazil unambiguously to a single region, and thus plotted these individuals using the mid-point co-ordinates of each country. No species were assigned to Northern Venzuela–Colombia, the Amazonia, the Cerrado or Coastal Brazil. We further looked at the distribution of fragrance groups across the eight biogeographic regions. We identified four fragrance groups present in Mesoamerica, seven in the Ithsmian region, eight in the northern Andes and four in the southern Andes. Table 2 summarizes the distribution of species and fragrance groups across the nine regions. In general, fragrance groups were not constrained to or clustered within a single region, but rather were dispersed across regions, with nine out of 15 fragrance groups present in at least two regions. In a few cases more than one species of the same fragrance group was present in the same biogeographic region.

**Table 2.** Distribution of fragrance groups and species across eight biogeographic regions and plotted in Fig. 4

Biogeographic region	Fragrance group	Species
Mesoamerica	7	*G.* aff. *unicolor*; *G. leuchochila*
	8	*G.* aff. *unicolor*
	9	*G.* aff. *unicolor*
	10	*G. cassidea*; *G. galeata*; *G. truncata*
Ithsmian	4	*G.* sp. chemotype A
	5	*G.* sp. chemotype S; *G.* aff. *odoratissma*
	6	*G.* sp. chemotype M; *G.* aff. *quinquenervis*
	8	*G. clavidora*
	10	*G.* aff. *quinquenervis*
	11	*G. fulva*
Northern Andes	1	*G. aromatica*
	2	*G. pleiochroma*
	3	*G. superflua*
	5	*G. aceras*
	6	*G. tracyana*
	11	*G. tricolor*
	12	*G. chocoensis*
	13	*G. ilense*; *G. scaphephorus*; *G. sphaerica*
	15	*G. grossa*
Southern Andes	1	*G. cruciformis*; *G. pleiochroma*
	2	*G. pleiochroma*
	3	*G. pleiochroma*
	13	*G. rufescens*
	14	*G. gracilis*
Northern Venezuela–Colombia	N/A	N/A
Amazonia	N/A	N/A
Cerrado	N/A	N/A
Coastal Brazil	N/A	N/A
Unassigned	8	*G.* sp. (Brazil)
	9	*G.* aff. *gracilis* (Venezuela)

**Fig. 4.**
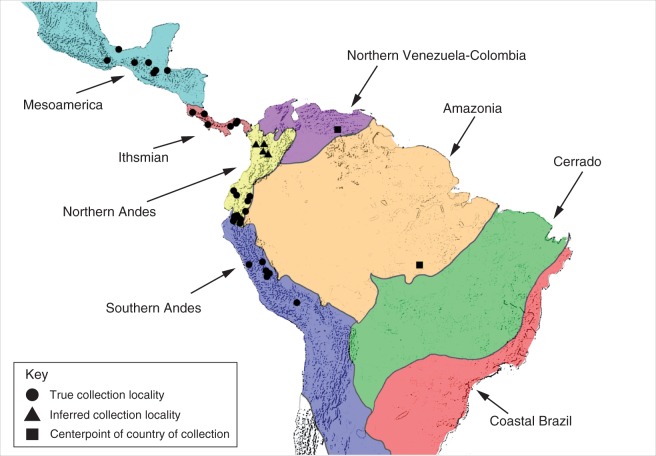
Depiction of the eight biogeographic regions as described by Gentry (1982). Points indicate the collection localities of all sampled (79) individuals for which we obtained scent profile data. Circles represent the true collection localities, whereas triangles represent inferred collection localities based on species distributions and type localities (Jenny, 1993). Squares represent the mid-point of the country of collection and are used for two individuals for which precise locality data were not available; these two individuals could not be unambiguously assigned to a single biogeographic region. Table 2 provides the distribution of fragrance groups and species within each biogeographic region.

## DISCUSSION

The genus *Gongora* exhibits specialized associations with male euglossine bees, in which male bees visit and pollinate orchid flowers while collecting and accumulating species-specific perfume compounds. Floral scent strongly modulates the architecture of plant–pollinator networks in this mutualism. However, a comprehensive study of the floral scent chemistry across the group has been largely missing. We conducted a comparative analysis of floral scent chemistry based upon 78 individuals belonging to 28 taxonomically described species from two of the three major lineages with samples derived from across the entire geographic range of *Gongora*. This roughly encompasses 35–45 % of the known species diversity of *Gongora*. We discuss: (1) the diversity of floral scent compounds and the patterns of species specificity; (2) the potential biological role that scent molecules play in regulating pollinator attraction and specialization; (3) the evidence for cryptic diversity as revealed by chemical traits; and (4) the distribution of floral scent compounds across taxonomic rank and geography.

### Floral scent diversity

We detected a total of 83 floral volatiles across *Gongora* species that were almost exclusively terpenes and aromatic compounds. Terpenes and aromatic compounds are derived from three well-conserved metabolic pathways present in all land plants (Chen *et al.*, 2011; Dudareva *et al.*, 2013). As such, many of the compounds we identified in *Gongora* are common across diverse angiosperm families and exhibit a high degree of functional diversity. For example, the terpenes β-ocimene, linalool and β-pinene, which were among the most prevalent compounds produced by *Gongora*, are commonly associated with the attraction of generalist pollinators including bees, flies and butterflies (Dobson, 2006). The broad phylogenetic distribution and functional diversity of floral volatiles produced by *Gongora* supports the hypothesis that regulatory changes controlling the production and localization of volatiles in orchid flowers probably resulted from the co-option of pre-existing ancestral metabolic pathways and molecular machineries (i.e. terpene synthase genes) that then facilitated the origin, subsequent diversification and specialization of this mutualism.

On the other hand, our analysis revealed the production of some compounds that are relatively rare outside of eulgossine bee-pollinated Orchidaceae (e.g. dimethyoxybenzene, *cis*-methyl *p*-methoxycinnamate, ipsdineol, chavicol, anethole, isoeugenol and 5-indanol). Among these, dimethyoxybenzene, *cis*-methyl *p*-methoxycinnamate, ipsdineol and isoeugenol are known to elicit collection behaviour in male bees. Following a similar pattern, compound #51 is probably restricted to orchids, but is a dominant component of the floral scent of three *Gonogra* species (*G. aceras*, *G.* aff. *odoratissma* and *G.* sp. chemotype S)*.* Interestingly, this same compound has been detected in the hind-leg extracts of several bee species from Panama, Costa Rica and Mexico (T. Eltz, pers. comm.), suggesting that male bees actively collect it. The presence of these phylogenetically restricted compounds suggests that the underlying metabolic pathways that control their production may have evolved *de novo* in *Gongora* and/or other euglossine bee-pollinated orchid lineages, probably in response to pre-existing sensory biases of male euglossine bees.

### Biological role of scent molecules

Because *Gongora* orchids depend exclusively on male euglossine bees for sexual reproduction, the overall chemical composition of floral scent is likely to be under strong selective pressure in response to the olfactory preferences of male euglossine bees. Field behavioural assays using synthetic forms of commonly identified floral volatiles produced by euglossine bee-pollinated orchids have revealed the presence of biologically active compounds that consistently attract and elicit collection behaviour in male euglossine bees (Dodson *et al.*, 1969; Williams and Whitten, 1983; Ackerman, 1989). We identified 29 attractive compounds produced by *Gongora*, including 1,8-cineole, eugenol and methyl salicylate. Contrary to our expectation, however, attractive compounds were not consistently among the most prevalent compounds nor were they emitted in high concentrations. Paradoxically, those compounds commonly regarded as poor attractants, including β-ocimene, linalool and α-farnesene (Williams and Dodson, 1972; Williams and Whitten, 1983; Ackerman, 1989), were among the most prevalent compounds and, in some cases, contributed up to 75 % of the floral scent bouquet in *Gongora*. Similar patterns have been observed in other euglossine bee-pollinated orchid genera including *Stanhopea*, *Cynoches* and *Catesetum* (Hills *et al.*, 1972; Gregg, 1983; Williams and Whitten, 1983)*.* The presence of these compounds may simply reflect the fact that they are widespread across diverse angiosperm families, are selectively neutral and/or perhaps represent enzymatic by-products or ancestral traits with little influence on bee behaviour or plant fitness. However, this may not apply to floral volatiles that are produced in high relative abundances given the potentially high metabolic cost of terpene synthesis (Gershenzon, 1994).

Multiple alternative explanations may reconcile the presence of poorly attractive compounds in the floral scent of *Gongora*. Field experiments have shown that poor attractants (including β-ocimene) in combination with strong attractants such as 1,8-cineole, eugenol and methyl salicylate reduce the luring potential of the attractive molecule, either by decreasing the number of individual bees or by lowering the number of bee species attracted (Williams and Dodson, 1972). These experiments revealed that non-attractive compounds function as behavioural modifiers that filter out certain species of bee pollinators. Such pollinator specialization contributes to the fine partitioning of bee communities and could have strong impacts on plant fitness and reproductive isolation. Alternatively, floral scents may have evolved through opposing selective pressures (Strauss and Whittall, 2006; Kessler and Halitschke, 2009), and some scent compounds may act as anti-herbivore defences. In fact, we have observed high levels of florivory by chrysomelid beetles in *Gongora* from Costa Rica (S. R. Ramirez and M. C. Hetherington-Rauth, pers. obs.), suggesting that herbivory may act as a potential selective agent. In sum, the role of each floral volatile remains unknown, and floral volatiles may experience multiple selective forces and contribute differentially to pollinator attraction and plant fitness. Manipulative experiments are needed to understand fully the role of scent traits, and neurophysiological approaches may reveal how the olfactory system of bees shaped floral scent traits (Milet-Pinheiro *et al.*, 2015).

### Cryptic diversity and specificity of scent bouquets

Our multivariate analysis revealed lower within-species variation relative to between-species variation in scent chemistry. In addition, it showed that scent profiles are largely species specific and revealed several potential cases of cryptic diversity among morphologically indistinguishable taxa.

We identified three distinct chemotypes in *Gongora pleiochroma* that could not be differentiated on the basis of geography or morphology. The variation in scent chemistry was explained by quantitative rather than qualitative differences in the chemical composition. Clusters were defined by the differential relative ratios of *cis*-β-ocimene, linalool and β-bisabolene. At this time, pollinator data are not available for *G. pleiochroma*. If different chemotypes were to attract similar pollinator assemblages, then the observed quantitative variation may simply reflect natural standing variation. In fact, similar quantitative variation in floral volatiles has been observed among populations of *Gongora* from central Panama that were visited by the same pollinator assemblage (Whitten, 1985). On the other hand, chemotype variation may reflect an adaptation to the dynamic preferences of male euglossine bees due to geographical and/or seasonal differences in the availability of different sources of volatiles (Whitten, 1985). Under the learned avoidance model, male euglossine bees display a satiation behaviour, and cease to collect a given volatile in favour of another in order to ensure the collection of species-specific perfume blends (Eltz *et al.*, 2006). Furthermore, recent studies have documented differences in the relative abundance of compounds in the hind-leg extracts of male bees due to seasonal and habitat differences depending on when and where the bees were collected, suggesting that volatiles may be temporally and/or spatially limited (Pokorny *et al.*, 2013; Eltz *et al.*, 2015). In the same study, when presented with pure synthetic compounds, male bees preferentially collected volatiles that were limited (Pokorny *et al.*, 2013). Thus the observed polymorphism in the floral scent of *G. pleiochroma* may represent an adaption for enhancing cross-pollination rates in the face of dynamic environmental conditions. Lastly, intraspecific variation may have resulted from pollinator-mediated directional selection if different species of male euglossine bees differentially visit each chemotype due to differences in innate olfactory preferences (Gregg, 1983). Under this scenario, intra-specific variation may persist over long evolutionary times, and could eventually lead to the formation of gene flow barriers.

Our analysis of the floral scent chemistry and pollinator network of La Gamba *Gongora* revealed an exceptional case of cryptic diversity. Individuals could not be differentiated on the basis of morphology, but segregated into distinct chemical phenotypes. We identified three chemotypes (A, M and S) based on the high relative abundances of *cis*-and *trans*-methyl *p*-methoxycinnamate, terpinen-4-ol and unidentified compound #51, respectively. Furthermore, volatile compounds were not shared among chemotypes, and our pollinator data indicate that each chemotype is visited by near non-overlapping sets of bee species. This raises the possibility that each chemotype is reproductively isolated and could probably represent cryptic species. Furthermore, all chemotypes occur in sympatry and bloom during the dry season (January–April), suggesting that geographic and temporal isolation do not pose barriers to gene flow. Our results support previous observations that floral scent, and not other floral traits (e.g. colour or flowering phenology) serves as the principal mechanisms of pollinator specificity (Dressler, 1966, 1968*b*)*.* Future studies may focus on quantifying the extent of genetic differentiation among chemotypes and experimentally measure hybrid fitness to examine the forces shaping scent evolution.

### Geographic and taxonomic distribution of floral scent phenotypes

Floral scent chemistry was not predicted by taxonomic rank or geography. Our INDVAL analysis revealed that floral volatiles were often shared among species of different subgenera and/or sections of *Gongora.* Similar floral scent phenotypes in unrelated lineages may represent convergent evolution, possibly driven by shared common olfactory preferences of male orchid bees. Although our data are limited to coarse taxonomic ranks, our findings support the previously proposed hypothesis that floral scent is an evolutionary labile trait (Knudsen *et al.*, 1993; Williams and Whitten, 1999; Levin *et al.*, 2003; Peakall *et al.*, 2010). In addition, the separation of morphologically indistinguishable species in chemical space suggests that scent traits evolve rapidly, possibly in response to pollinator-mediated selection.

The INDVAL analysis also revealed that floral scent phenotypes are broadly distributed across biogeographic regions, and do not exhibit a spatially clustered pattern. The mechanism that led to this pattern remains unknown, but a possible scenario is that scent phenotypes diverged through reproductive character displacement in response to selection against lower fitness hybrids or higher costs of hybrid mating. In this scenario, co-occurring species of *Gongora* may be selected to display divergent scent phenotypes, thus enabling fine partitioning of local bee pollinators. On the other hand, we observed few cases where multiple taxa of the same fragrance group occupy the same biogeographic region. We speculate that these lineages may coexist either because minor floral scent differences lead to fine partitioning of bee pollinators or instead because ecological differences between taxa contribute to allopatric distributions (Dodson 1978). However, the precise distribution of most *Gongora* taxa remains uncertain (Jenny 1993).

### Concluding remarks

Plant–pollinator mutualisms are striking examples of coevolution. However, efforts to understand how mutualisms originate and persist through evolutionary time have been hampered by the complexity of most species interactions and the difficulty in isolating important traits. Floral scent is an important component of the trait repertoire that flowering plants use to attract, select and manipulate animal pollinators. However, despite the ubiquitous distribution of floral scent across the angiosperm phylogeny, scent traits have received limited attention. Our study takes advantage of a simple and powerful chemical signalling system of pollinator attraction, and provides the first extensive characterization of the diversity and variation of floral scent chemistry across the genus *Gongora*. We present evidence consistent with the hypothesis that floral scent in *Gongora* is largely species specific and divergent among related and sympatric lineages. Furthermore, we provide integral information for future experimental manipulations that could facilitate linking phenotypic trait variation to pollinator specificity and, potentially, genetic divergence. Future studies may focus on elucidating the molecular mechanisms that control pollinator specialization in this group by studying the terpene biosynthetic pathway (TPS enzymes).

## SUPPLEMENTARY DATA

Supplementary data are available online at www.aob.oxfordjournals.org and consist of the following. Table S1: sampled taxon groups. Table S2: accession numbers of sampled individuals. Table S3: Excel spreadsheet of the average relative abundance and standard deviation of floral volatiles for (A) each sampled individual averaged across sample replicates and (B) each species averaged across the mean of individuals. Figure S1: *Gongora* species belonging to subgenus *Acropera* sections *Acropera* and *Cassidea*. Figures S2–S4: *Gongora* species belonging to subgenus *Gongora* section *Gongora*. Figures S5 and S6: *G. pleiochroma* arranged by fragrance groups. Figure S7: *Gongora* species from the population La Gamba, Costa Rica. Figure S8: *Gongora* species belonging to subgenus *Gongora* sections *Grossa* and *Truncata.* Figure S9: clustering dendrogram based on the Bray–Curtis dissimilarity metric.

Supplementary Data
