# Supplementary material for: Evolution and diversity of floral scent chemistry in the euglossine bee-pollinated orchid genus Gongora
Source: Ann Bot. 2016 May 30;118(1):135–48. doi: 10.1093/aob/mcw072 (PMC4934395; doi:10.1093/aob/mcw072)
Supplement: Supplementary Data [file supp_118_1_135__index.html]

Evolution and diversity of floral scent chemistry in the euglossine bee-pollinated orchid genus Gongora — Supplementary Data 

# Evolution and diversity of floral scent chemistry in the euglossine bee-pollinated orchid genus *Gongora*

## Supplementary Data

files

- Supplementary Data - zip file
