# Supplementary material for: Evolution and diversity of floral scent chemistry in the euglossine bee-pollinated orchid genus Gongora
Source: Ann Bot. 2016 May 30;118(1):135–48. doi: 10.1093/aob/mcw072 (PMC4934395; doi:10.1093/aob/mcw072)
Supplement: Supplementary Data [file supp_mcw072_suppl_data.zip › aob-15945-s03.pdf]

Supplemental Table 1—

Table of sampled species. Species are arranged according to their hierarchical taxonomic rank. The total individuals sampled indicate the total number of individuals for the given species that were sampled and that produced resolvable chromatograms. Numbers in parenthesis represent the number of individuals that we sampled but which did not produce resolvable chromatograms; number not included in total.

Taxonomic classification	Total Individuals Sampled
**Subgenus Portentosa**	
**Subgenus Acropera**	
**Section *Acropera***	
G. galeata Lindley	3
**Section *Armeniaca***	
**Section *Cassidea***	
G. cassidea Rchb.f.	2
**Subgenus Gongora**	
**Section *Gongora***	
G. aceras Dressler	2
G. aromatica Rchb.f.	1
G. chocoensis Jenny	1 (1)
G. clavidora Dressler	1
G. cruciformis Whitten & D.E.Benn	1
G. fulva Lindley	2
G. gracilis Jenny	3
G. ilense Whitten & Jenny	1
G. leucochila Lemaire	1 (2)
G. aff. *odoratissma* Lemaire	1
G. pleiochroma Rchb.f.	17 (3)
G. powellii Schlechter	7
G. aff. *quinquenervis* Ruiz & Pavon	2
G. rufescens Jenny	1
G. sp. (Brazil)	1
G. sp. Chemotype A	6
G. sp. Chemotype M	4
G. sp. Chemotype S	2
G. superflua Rchb.f.	1 (2)
G. tricolor [Lindley] Rchb.f.	1
G. aff. *unicolor* Schlechter	6 (2)
**Section *Gratulabunda***	
**Section *Grossa***	
G. grossa Rchb.f.	5
**Section Truncata**	
G. scaphephorus Rchb.f. & Warsc.	1
G. sphaerica Jenny	2
G. tracyana Rolfe	2
G. truncata Lindley	1
