# Supplementary material for: Evolution and diversity of floral scent chemistry in the euglossine bee-pollinated orchid genus Gongora
Source: Ann Bot. 2016 May 30;118(1):135–48. doi: 10.1093/aob/mcw072 (PMC4934395; doi:10.1093/aob/mcw072)
Supplement: Supplementary Data [file supp_mcw072_suppl_data.zip › aob-15945-s04.pdf]

Supplemental Table 2—

Table of accession numbers of sampled individuals. N refers to the number of samples collected that produced high resolution chromatograms; these samples were included in the dataset used for analysis. When N is zero it indicates that the individual was sampled but that the samples did not produced resolvable chromatograms. GCMS Method refers to the injection split ratio used for analysis to produce high-resolution chromatograms. Biogeographic region refers to the regions depicted in Figure 4. Abbreviation: ABG=Atlantic Botanical Garden

Living UCD Accession Number	Subgenus	Section	Species	Chemotype	N	GCMS Method	Collection Locality	Biogeographic Region	Acquired From
UCD-0049	*Acropera*	*Acropera*	*G. galeata* Lindley		2	split 5:1	Laguna Ocotalito in Montes Azules Natural Park, Chiapas, Mexico	Mesoamerica	M. Whitten
UCD-0053	*Acropera*	*Acropera*	*G. galeata* Lindley		4	split 5:1	Oaxaca, Mexico	Mesoamerica	M. Whitten
UCD-0054	*Acropera*	*Acropera*	*G. galeata* Lindley		3	split 5:1	Oaxaca, Mexico	Mesoamerica	M. Whitten
UCD-0038	*Acropera*	*Cassidea*	*G. cassidea* Rchb.f		2	split 5:1	Alta Verapaz, Guatemala	Mesoamerica	M. Whitten
UCD-0040	*Acropera*	*Cassidea*	*G. cassidea* Rchb.f		2	split 5:1	Guatemala	Mesoamerica	M. Whitten
UCD-0032	*Gongora*	*Gongora*	*G. aceras* Dressler		2	split 5:1	Colombia[1]	Northern Andes	M. Whitten
UCD-0033	*Gongora*	*Gongora*	*G. aceras* Dressler		1	split 5:1	Colombia	Northern Andes	M. Whitten
UCD-0030	*Gongora*	*Gongora*	*G. aromatica* Rchb.f.		4	split 5:1	Along Río Jamboe, Zamora, Ecuador	Northern Andes	M. Whitten
UCD-0174	*Gongora*	*Gongora*	*G. chocoensis* Jenny		2	splitless	NA[2]	Northern Andes	ABG
UCD-0189	*Gongora*	*Gongora*	*G. chocoensis* Jenny		0	Unresolvable	NA[2]	Northern Andes	ABG
UCD-0043	*Gongora*	*Gongora*	*G. clavidora* Dressler		6	split 5:1	Los Angeles Norte, Alujuela, Costa Rica	Costa Rica & Panama	M. Whitten
UCD-0046	*Gongora*	*Gongora*	*G. cruciformis* Whitten & D.E.Benn		5	split 5:1	Oxapampa, Peru	Southern Andes	M. Whitten
UCD-0184	*Gongora*	*Gongora*	*G. fulva* Lindley		4	split 5:1	Ecuador	Northern Andes	ABG
UCD-0199	*Gongora*	*Gongora*	*G. fulva* Lindley		2	split 5:1	Regions of Esmeraldas & Manabi, Ecuador	Northern Andes	Ecuagenera Nursery
UCD-0023	*Gongora*	*Gongora*	*G. gracilis* Jenny[3]		3	split 5:1	Leonicio Prado, Peru	Southern Andes	M. Whitten
UCD-0063	*Gongora*	*Gongora*	*G. gracilis* (Venezuela) Jenny		2	splitless	Venezuela	?	M. Whitten
UCD-0066	*Gongora*	*Gongora*	*G. gracilis* Jenny		1	splitless	Tingo Maria, Leonicio Prado, Peru	Southern Andes	M. Whitten
UCD-0081	*Gongora*	*Gongora*	*G. ilense* Whitten & Jenny		2	split 5:1	Centinela, Sucumbíos, Ecuador	Northern Andes	M. Whitten
UCD-0086	*Gongora*	*Gongora*	*G. leucochila* Lemaire		0	Unresolvable	Laguna Escondida, Veracruz, Mexico	Mesoamerica	M. Whitten
UCD-0087	*Gongora*	*Gongora*	*G. leucochila* Lemaire		5	splitless	Laguna Escondida, Veracruz, Mexico	Mesoamerica	M. Whitten
UCD-0089	*Gongora*	*Gongora*	*G. leucochila* Lemaire		0	Unresolvable	Laguna Escondida, Veracruz, Mexico	Mesoamerica	M. Whitten
UCD-0091	*Gongora*	*Gongora*	*G.* sp. Lindley		5	split 5:1	Brazil	?	M. Whitten
UCD-0093	*Gongora*	*Gongora*	*G.* aff. *odoratissma* Lemaire[4]		5	split 5:1	Guapiles, Limón, Costa Rica	Costa Rica & Panama	M. Whitten
UCD-0003	*Gongora*	*Gongora*	*G. pleiochroma*	linalool	1	split 5:1	Puyo, Pastaza, Ecuador		M. Whitten

			Rchb.f.						
UCD-0004	*Gongora*	*Gongora*	*G. pleiochroma* Rchb.f.	linalool	4	split 5:1	Mendez, Morona Santiago, Ecuador	Northern Andes	M. Whitten
UCD-0006	*Gongora*	*Gongora*	*G. pleiochroma* Rchb.f.	unknown	0	unresolvable	no locality	?	M. Whitten
UCD-0008	*Gongora*	*Gongora*	*G. pleiochroma* Rchb.f.	linalool	4	split 5:1	Río Pozuzo, Oxapampa, Peru	Southern Andes	M. Whitten
UCD-0010	*Gongora*	*Gongora*	*G. pleiochroma* Rchb.f.	linalool	1	split 5:1	Hacienda Santa Clara, Chanchamayo, Peru	Southern Andes	M. Whitten
UCD-0011	*Gongora*	*Gongora*	*G. pleiochroma* Rchb.f.	E-beta ocimene	5	split 5:1	no locality	?	M. Whitten
UCD-0013	*Gongora*	*Gongora*	*G. pleiochroma* Rchb.f.	linalool	1	split 5:1	Puerto Yurinak, Chanchamayo, Peru	Southern Andes Southern Andes	M. Whitten
UCD-0014	*Gongora*	*Gongora*	*G. pleiochroma* Rchb.f.	beta bisabolene	2	split 5:1	Peru	Southern Andes	M. Whitten
UCD-0015	*Gongora*	*Gongora*	*G. pleiochroma* Rchb.f.	unknown	0	unresolvable	Mendez along Río Paute,Morona Santiago, Ecuador	Northern Andes	M. Whitten
UCD-0016	*Gongora*	*Gongora*	*G. pleiochroma* Rchb.f.	linalool	4	split 5:1	Puerto Napo, Napo, Ecuador	Northern Andes	M. Whitten
UCD-0017	*Gongora*	*Gongora*	*G. pleiochroma* Rchb.f.	E-beta ocimene	2	split 5:1	Río Pozuzo, Oxapampa, Peru	Southern Andes	M. Whitten
UCD-0018	*Gongora*	*Gongora*	*G. pleiochroma* Rchb.f.	linalool	1	split 5:1	El Progreso near Río Yacuamba, Zamora-Chinchipe, Ecuador	Northern Andes	M. Whitten
UCD-0019	*Gongora*	*Gongora*	*G. pleiochroma* Rchb.f.	beta bisabolene	4	split 5:1	Peru		M. Whitten
UCD-0020	*Gongora*	*Gongora*	*G. pleiochroma* Rchb.f.	E-beta ocimene	4	split 5:1	Puerto Bermudez, Oxapampa, Peru	Southern Andes	M. Whitten
UCD-0021	*Gongora*	*Gongora*	*G. pleiochroma* Rchb.f.	linalool	2	split 5:1	Río Pozuzo, Oxapampa, Peru	Southern Andes	M. Whitten
UCD-0022	*Gongora*	*Gongora*	*G. pleiochroma* Rchb.f.	E-beta ocimene	1	split 5:1	Río Pozuzo, Oxapampa, Peru	Southern Andes	M. Whitten
UCD-0024	*Gongora*	*Gongora*	*G. pleiochroma* Rchb.f.	linalool	6	split 5:1	Moyobamba, Chanchamayo, Peru	Southern Andes	M. Whitten
UCD-0026	*Gongora*	*Gongora*	*G. pleiochroma* Rchb.f.	E-beta ocimene	2	split 5:1	Río Pozuzo, Oxapampa, Peru	Southern Andes	M. Whitten
UCD-0027	*Gongora*	*Gongora*	*G. pleiochroma* Rchb.f.	linalool	2	splitless	Puerto Napo, Ecuador	Northern Andes	M. Whitten
UCD-0163	*Gongora*	*Gongora*	*G. pleiochroma* Rchb.f.	uknown	0	unresolvable	Mendez along Río Paute, Morona Santiago, Ecuador	Northern Andes	M. Whitten
UCD-0035	*Gongora*	*Gongora*	*G. powellii* Schlechter[5]		3	split 5:1	El Valle de Anton, Coclé, Panama	Costa Rica & Panama	M. Whitten
UCD-0094	*Gongora*	*Gongora*	*G. powellii* Schlechter		2	split 5:1	El Valle de Anton, Coclé, Panama	Costa Rica & Panama	M. Whitten
UCD-0095	*Gongora*	*Gongora*	*G. powellii* Schlechter		3	split 5:1	El Valle de Anton, Coclé, Panama	Costa Rica & Panama	M. Whitten
UCD-0097	*Gongora*	*Gongora*	*G. powellii* Schlechter		9	split 5:1	El Valle de Anton, Coclé, Panama	Costa Rica & Panama	M. Whitten
UCD-0103	*Gongora*	*Gongora*	*G. powellii*		4	split 5:1	El Valle de Anton, Coclé, Panama	Costa Rica &	M. Whitten

			Schlechter					Panama	
UCD-0183	*Gongora*	*Gongora*	*G. powellii* Schlechter		2	split 5:1	El Valle de Anton, Coclé, Panama	Costa Rica & Panama	ABG
UCD-0186	*Gongora*	*Gongora*	*G. powellii* Schlechter		1	split 5:1	Río Iguanita near Portobelo, Colón, Panama	Costa Rica & Panama	ABG
UCD-0106	*Gongora*	*Gongora*	*G.* aff. *quinquenervis*		1	splitless	Costa Rica (possibly Guancaste)	Costa Rica & Panama	M. Whitten
UCD-0107	*Gongora*	*Gongora*	*G.* aff. *quinquenervis* Ruiz & Pavon[6]		2	split 5:1	Guancaste, Costa Rica	Costa Rica & Panama	M. Whitten
UCD-112	*Gongora*	*Gongora*	*G. rufescens* Jenny		4	split 5:1	Peru[7]	Southern Andes	M. Whitten
UCD-0091	*Gongora*	*Gongora*	*G.* sp. (Brazil)[8]		5	split 5:1	Brazil	?	M. Whitten
G-45B	*Gongora*	*Gongora*	*G.* sp.	A	2	split 5:1	La Gamba, Puntarenas, Costa Rica	Costa Rica & Panama	Field
G-49	*Gongora*	*Gongora*	*G.* sp.	A	1	split 5:1	La Gamba, Puntarenas, Costa Rica	Costa Rica & Panama	Field
G-56	*Gongora*	*Gongora*	*G.* sp.	A	2	split 5:1	La Gamba, Puntarenas, Costa Rica	Costa Rica & Panama	Field
G-64	*Gongora*	*Gongora*	*G.* sp.	A	1	split 5:1	La Gamba, Puntarenas, Costa Rica	Costa Rica & Panama	Field
G-95	*Gongora*	*Gongora*	*G.* sp.	A	1	split 5:1	La Gamba, Puntarenas, Costa Rica	Costa Rica & Panama	Field
G-97	*Gongora*	*Gongora*	*G.* sp.	A	1	split 5:1	La Gamba, Puntarenas, Costa Rica	Costa Rica & Panama	Field
G-1	*Gongora*	*Gongora*	*G.* sp.	M	1	split 5:1	La Gamba, Puntarenas, Costa Rica	Costa Rica & Panama	Field
G-24	*Gongora*	*Gongora*	*G.* sp.	M	1	split 5:1	La Gamba, Puntarenas, Costa Rica	Costa Rica & Panama	Field
G-57	*Gongora*	*Gongora*	*G.* sp.	M	1	split 5:1	La Gamba, Puntarenas, Costa Rica	Costa Rica & Panama	Field
G-59	*Gongora*	*Gongora*	*G.* sp.	M	1	split 5:1	La Gamba, Puntarenas, Costa Rica	Costa Rica & Panama	Field
G-17	*Gongora*	*Gongora*	*G.* sp.	S	1	split 5:1	La Gamba, Puntarenas, Costa Rica	Costa Rica & Panama	Field
G-61	*Gongora*	*Gongora*	*G.* sp.	S	1	split 5:1	La Gamba, Puntarenas, Costa Rica	Costa Rica & Panama	Field
UCD-0123	*Gongora*	*Gongora*	*G. superflua* Rchb.f.		4	split 5:1	Cuenca, El Oro, Ecuador	Northern Andes	M. Whitten
UCD-0124	*Gongora*	*Gongora*	*G. superflua* Rchb.f.		0	unresolvable	Cuenca, El Oro, Ecuador	Northern Andes	M. Whitten
UCD-0182	*Gongora*	*Gongora*	*G. superflua* Rchb.f.		0	unresolvable	Chilla, El Oro, Ecuador	Northern Andes	ABG
UCD-0127	*Gongora*	*Gongora*	*G. tricolor* [Lindl] Rchb.f.		1	split 5:1	Cerro Azul, Panama, Panama	Costa Rica & Panama	M. Whitten
UCD-0133	*Gongora*	*Gongora*	*G.* aff. *unicolor* Schlechter		1	splitless	along Río Dulce, Izabal, Guatemala	Mesoamerica	M. Whitten
UCD-0134	*Gongora*	*Gongora*	*G.* aff. *unicolor* Schlechter		2	splitless	Laguna Escondia, Veracruz, Mexico	Mesoamerica	M. Whitten
UCD-0135	*Gongora*	*Gongora*	*G.* aff. *unicolor* Schlechter		0	Unresolvable	Laguna Escondia, Veracruz, Mexico	Mesoamerica	M. Whitten
UCD-0136	*Gongora*	*Gongora*	*G.* aff. *unicolor* Schlechter		2	splitless	Laguna Escondia, Veracruz, Mexico	Mesoamerica	M. Whitten

UCD-0137	*Gongora*	*Gongora*	*G.* aff. *unicolor* Schlechter	2	split 5:1	Bonampak, Chiapas, Mexico	Mesoamerica	M. Whitten
UCD-0139	*Gongora*	*Gongora*	*G.* aff. *unicolor* Schlechter	1	splitless	Bonampak, Chiapas, Mexico	Mesoamerica	M. Whitten
UCD-0140	*Gongora*	*Gongora*	*G.* aff. *unicolor* Schlechter	0	Unresolvable	Bonampak, Chiapas, Mexico	Mesoamerica	M. Whitten
UCD-0141	*Gongora*	*Gongora*	*G.* aff. *unicolor* Schlechter	1	splitless	Bonampak, Chiapas, Mexico	Mesoamerica	M. Whitten
UCD-0028	*Gongora*	*Grossa*	*G. grossa* Rchb.f.[9]	4	split 5:1	Mendez, Morona-Santiago, Ecuador	Northern Andes	M. Whitten
UCD-0069	*Gongora*	*Grossa*	*G. grossa* Rchb.f.	3	split 5:1	Zaruma, El Oro, Ecuador	Northern Andes	M. Whitten
UCD-0074	*Gongora*	*Grossa*	*G. grossa* Rchb.f.	1	split 5:1	Buenavista, El Oro, Ecuador	Northern Andes	M. Whitten
UCD-0075	*Gongora*	*Grossa*	*G. grossa* Rchb.f.	2	split 5:1	Buenavista, El Oro, Ecuador	Northern Andes	M. Whitten
UCD-0076	*Gongora*	*Grossa*	*G. grossa* Rchb.f.	1	split 5:1	Zaruma, El Oro, Ecuador	Northern Andes	M. Whitten
UCD-0114	*Gongora*	*Truncata*	*G. scaphephorus* Rchb.f. & Warsc.	1	split 5:1	Cordillera de Cutucu near Mendez, Morona-Santiago, Ecuador	Northern Andes	M. Whitten
UCD-0119	*Gongora*	*Truncata*	*G. sphaerica* Jenny	2	split 5:1	Colombia	Northern Andes	M. Whitten
UCD-0120	*Gongora*	*Truncata*	*G. sphaerica* Jenny	1	split 5:1	Colombia	Northern Andes	M. Whitten
LS-1	*Gongora*	*Truncata*	*G. tracyana* Rolfe	1	split 5:1	Colombia[10]	Northern Andes	Mario Landoño
LS-2	*Gongora*	*Truncata*	*G. tracyana* Rolfe	1	split 5:1	Colombia	Northern Andes	Mario Landoño
UCD-0157	*Gongora*	*Truncata*	*G. truncata* Lindley	2	split 5:1	Laguna Escondia, Veracruz, Mexico	Mesoamerica	M. Whitten

[1] The species *Gongora aceras* is found along the western slopes of the Andes mountain range in Colombia and Ecuador (Jenny, 1993; www.orchidspecies.com).
[2] The species *Gongora chocoensis* has a restricted range to western Colombia (www. orchidspecies.com).
[3] Individual was acquired identified as *Gongora pleiochroma.* The species identification was changed to *Gongora gracilis* as inferred from the morphology of the labellum and scent profile dominated by indole similar to other *Gongora gracilis* sampled. Locality information remains consistent.
[4] We grouped this individual with *Gongora* species chemotype S collected from the Esquinas Rainforest in Costa Rica for data analysis which required discrete taxon groupings.
[5] Individual was acquired identified as *Gongora borucayensis.* The species identification was changed to *Gongora powellii* as inferred from the morphology and coloration of the flower and the scent profile characterized by (E)-beta ocimene and cis- and trans- MMC similar to other *Gongora powellii* sampled. Furthermore the locality information is consistent with other individuals named *G. powellii*.
[6] We grouped this individual with *Gongora* species chemotype A collected from the Esquinas Rainfores in Costa Rica for data analysis which requied discrete taxon groupings.
[7] The species *Gongora rufescens* is found in wet montane forests of Colombia, Ecuador, and Peru (www.orchidspeices.com).
[8]Individual was acquired identified as *Gongora nigrita.* The species identification was changed to *G.* sp. (Brazil) because of the incongruence with the taxonomic description provided by Jenny (1993).
[9] Individual was acquired identified as *Gongora pleiochroma.* The species identification was changed to *Gongora grossa* as inferred from the morphology of the labellum and the scent profile characterized by 4-anisyl acetate and DMB similar to other *Gongora grossa* sampled. Locality information remains consistent.
[10] The species *Gongora tracyana* is known from Colombia. These individuals of *Gongora* cf. *tracyana* likely were collected from areas of western Colombia and the Andes Mountain Range, although locality data is not well known.
