# Supplementary material for: Evolution and diversity of floral scent chemistry in the euglossine bee-pollinated orchid genus Gongora
Source: Ann Bot. 2016 May 30;118(1):135–48. doi: 10.1093/aob/mcw072 (PMC4934395; doi:10.1093/aob/mcw072)
Supplement: Supplementary Data [file supp_mcw072_suppl_data.zip › aob-15945-s06.docx]

**Supplemental Material**

**Supplemental Table 1—**

Table of sampled taxon groups. Taxon groups are arranged according to their hierarchical taxonomic groupings. The total individuals sampled indicate the total number of individuals for the given species that were sampled and that produced resolvable chromatograms. Numbers in parenthesis represent the number of individuals that we sampled but which did not produce resolvable chromatograms; number not included in total.

**Supplemental Table 2—**

Table of accession numbers of sampled individuals. N refers to the number of samples collected that produced high resolution chromatograms; these samples were included in the dataset used for analysis. When N is zero it indicates that the individual was sampled but that the samples did not produced resolvable chromatograms. GCMS Method refers to the injection split ratio used for analysis to produce high-resolution chromatograms. Biogeographic region refers to the regions depicted in Figure 4.

**Supplemental Table 3—**

Excel spreadsheet of the average relative abundance and standard deviation of floral volatiles for **A)** each sampled individual averaged across sample replicates and **B)** each species averaged across the mean of individuals. Compounds are grouped by chemical class and then listed in order of their retention time (RT). The Kovat’s Index (KI) was calculated using a series of alkane standards (C7-C30). Compound ID references the identifying number used in our local users library assembled throughout the duration of the study. The m/z ratios for the unidentified compounds are listed in order of increasing m/z ratio such that the molecular ion (the heaviest ion) is last. The base peak (the peak with the highest intensity) is indicated with an asterisk.

**Figure S1—**

*Gongora* species belonging to subgenus *Acropera* sections *Acropera* and *Cassidea*. **A,B)** *G. galeata.* **C)** *G. galeata* yellow morph. **D,E)** *G. cassidea*

**Figure S2—**

*Gongora* species belonging to subgenus *Gongora* section *Gongora*. **A,B)** *G. aceras.* **C)** *G. aromatica.* **D-F)** *G. chocensis.* **F)** *G. clavidora*. **G)** *G. cruciformis.* **H,I)** *G. fulva****.* J)** *Gongora* aff. *gracilis.* **K,L)** *G. gracilis.*

**Figure S3—**

*Gongora* species belonging to subgenus *Gongora* section *Gongora* continued. **A)** *G. ilense.* **B-D)** *G. leuchochila*. **E)** *G. nigrita.* **F)** *G.* aff. *odoratissma*. **G-M)** *G. powellii.*

**Figure S4—**

*Gongora* species belonging to subgenus *Gongora* section *Gongora* continued. **A,B)** *G.* aff. quinquenervis. **C)** *G. rufescens.* **D)** *G. nigrita.* **E, F)** *G. superflua*. **G)** *G. tricolor*. **H-I)** *G.* aff. *unicolor*.

**Figure S5—**

*Gongora* species belonging to subgenus *Gongora* section *Gongora* continued. *G. pleiochroma* arranged by fragrance groups. **A,B)** *G. pleiochroma* chemotype beta bisabolene. **C-L)** *G. pleiochroma* chemotype linalool.

**Figure S6—**

*Gongora* species belonging to subgenus *Gongora* section *Gongora* continued. *G. pleiochroma* arranged by fragrances groups. **A-E)** *G. pleiochroma* chemotype beta ocimene. **F-H)** *G. pleiochroma* unidentified chemotype.

**Figure S7—**

*Gongora* species belonging to subgenus *Gongora* section *Gongora* continued. *Gongora* species from the population La Gamba, Costa Rica. **A-F)** *Gongora* sp. chemotype A. **G-J)** *Gongora* sp. chemotype M. **K,L)** *Gongora* sp. chemotype S.

**Figure S8—**

*Gongora* species belonging to subgenus *Gongora* sections *Grossa* and *Truncata.* **A-E)** *G. grossa.* **F)** *G. scaphephorus.* **G- H)** *G. sphaerica*. **I)** *G. tracyana****.* J)** *G. truncata.*

**Figure S9—**

Clustering dendrogram based on the Bray-Curtis dissimilarity metric. With few exceptions, replicates of the same individual consistently clustered tightly together, indicating that both individual-level variation and instrument error were negligible relative to the variation observed between taxa. Replicates did not cluster by day after anthesis (either 1, 2, or 3) indicating that the overall scent chemistry remains constant during the lifetime of the inflorescence.
