# Supplementary material for: Evolution and diversity of floral scent chemistry in the euglossine bee-pollinated orchid genus Gongora
Source: Ann Bot. 2016 May 30;118(1):135–48. doi: 10.1093/aob/mcw072 (PMC4934395; doi:10.1093/aob/mcw072)
Supplement: Supplementary Data [file supp_mcw072_suppl_data.zip › aob-15945-s01.pdf]

Subgenus Acropera

Section *Acropera*

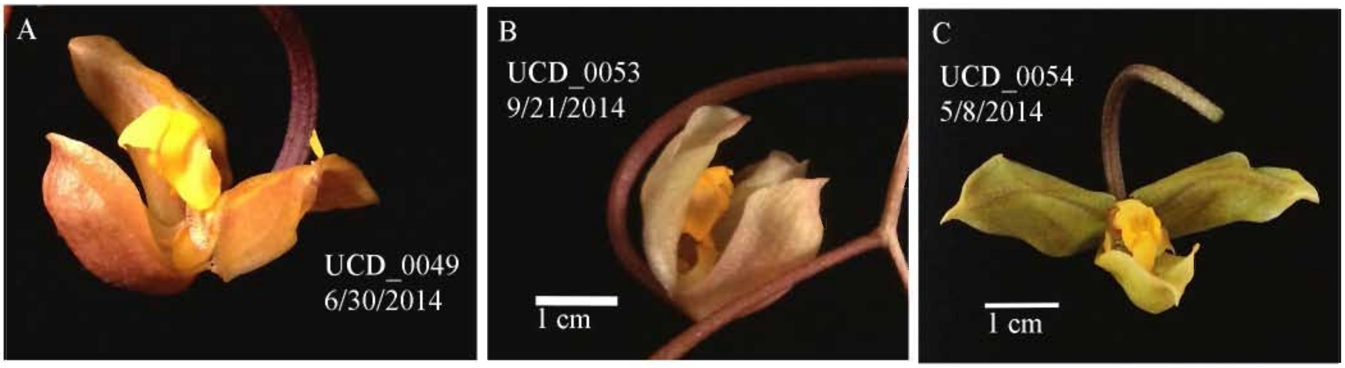

Section *Cassidea*

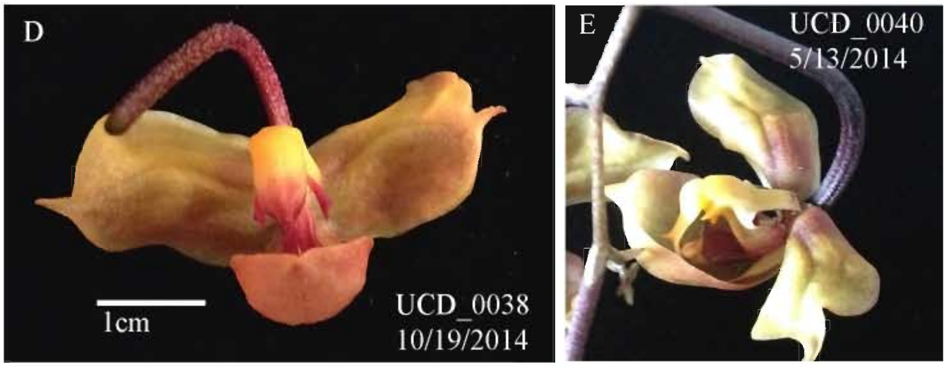

Figure S1: *Gongora* species belonging to Subgenus Acropera sections *Acropera* and *Cassidea*. **(A-B)** *Gongora galeata*. **(C)** *Gongora galeata* yellow morph. **(D-E)** *Gongora cassidea*.

Subgenus Gongora

Section *Gongora*

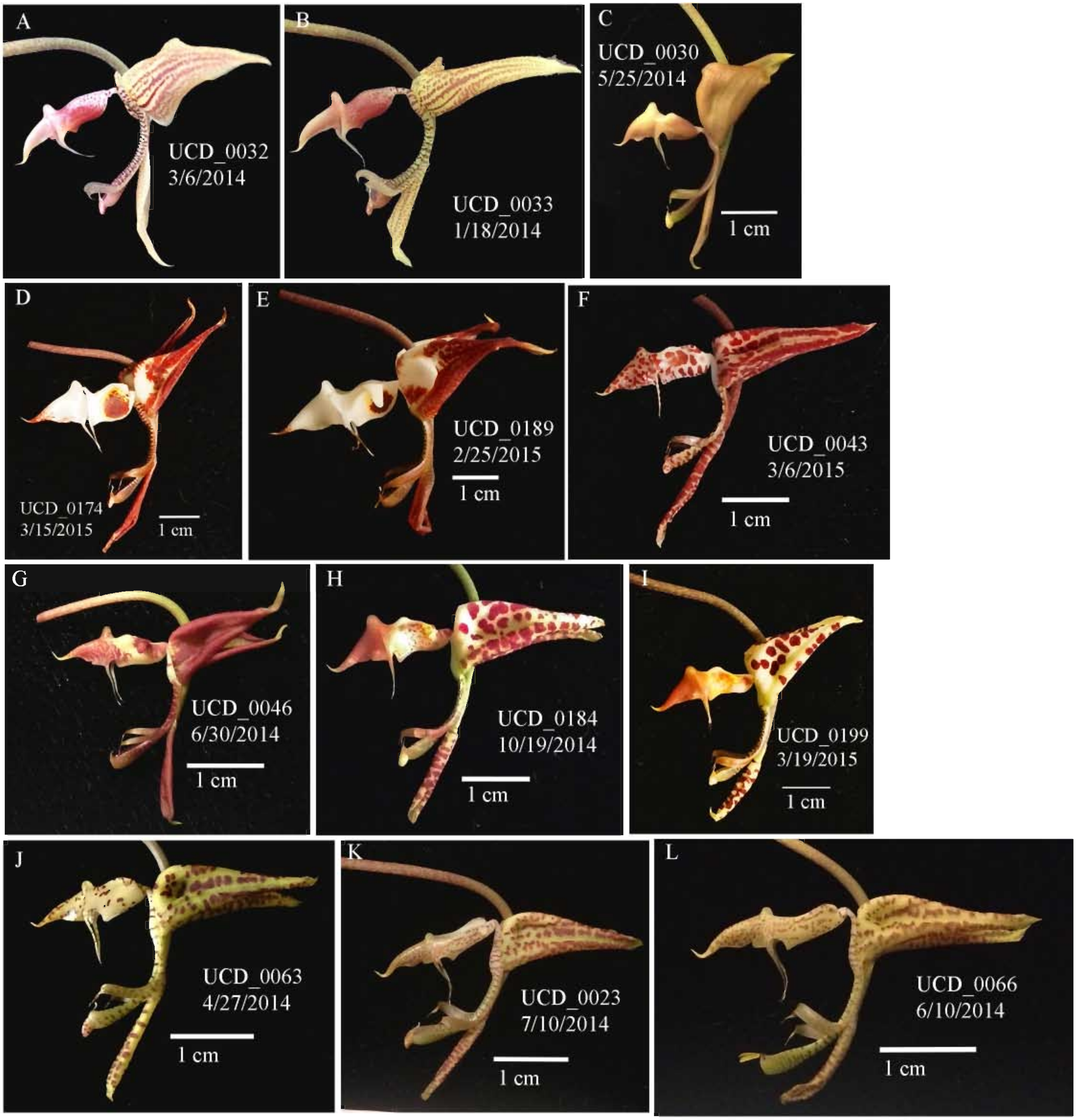

Figure S2: *Gongora* species belonging to Subgenus Gongora section section *Gongora*. (A,B) *Gongora aceras*. (C) *Gongora aromatica*. (D,E) *Gongora chocoensis*. (F) *Gongora clavidora*. (G) *Gongora cruciformis*. (H,I) *Gongora fulva*. (J) *Gongora* aff. *gracilis*. (K, L) *Gongora gracilis*.

Section *Gongora* continued

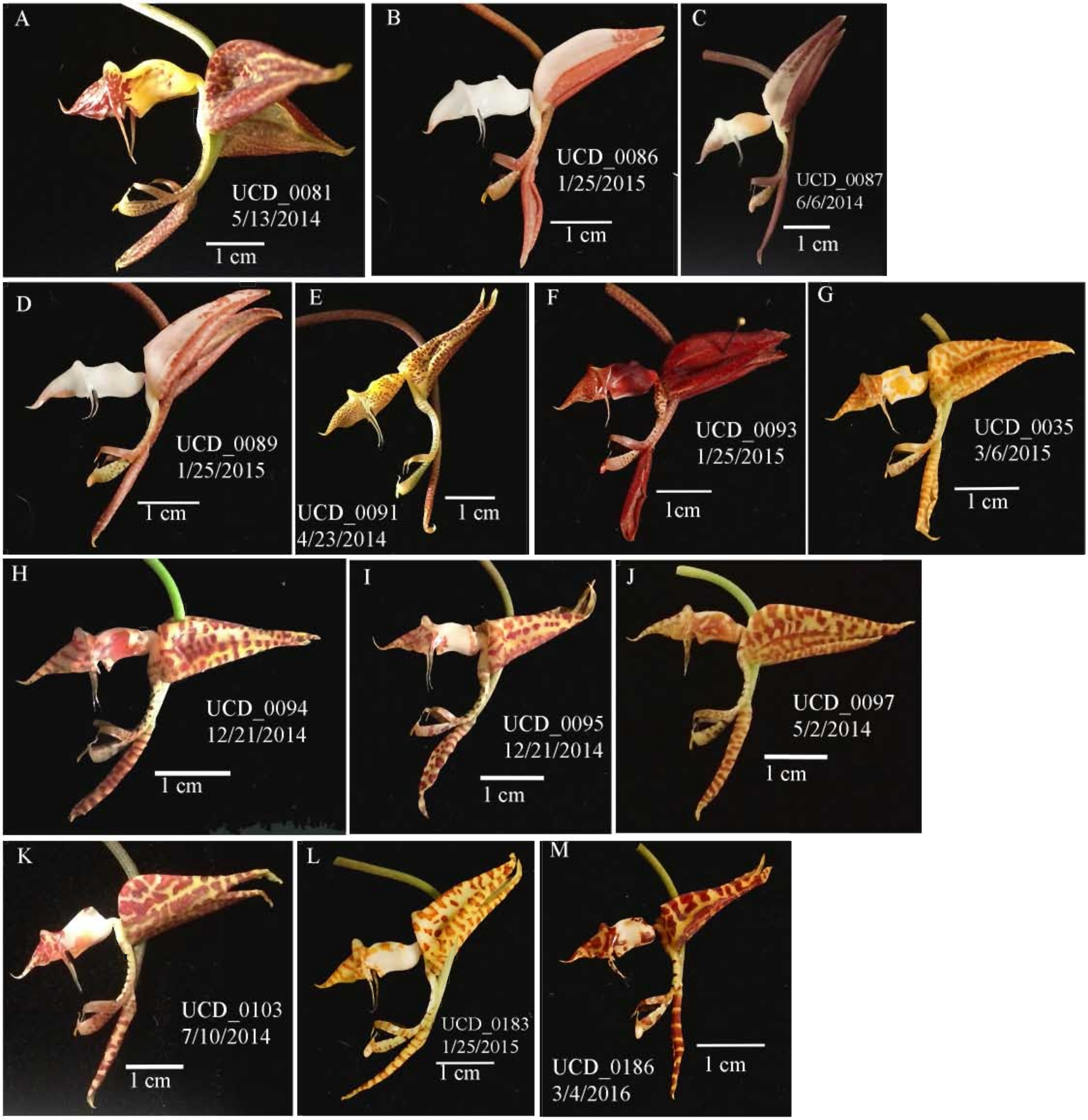

Figure S3: *Gongora* species belonging to Subgenus *Gongora* section *Gongora* continued. **(A)** *Gongora ilense.* **(B-D)** *Gongora leucochila.* **(E)** *Gongora nigrita.* **(F)** *Gongora aff. odoratissma.* **(G-M)** *Gongora powellii.*

Section *Gongora* continued

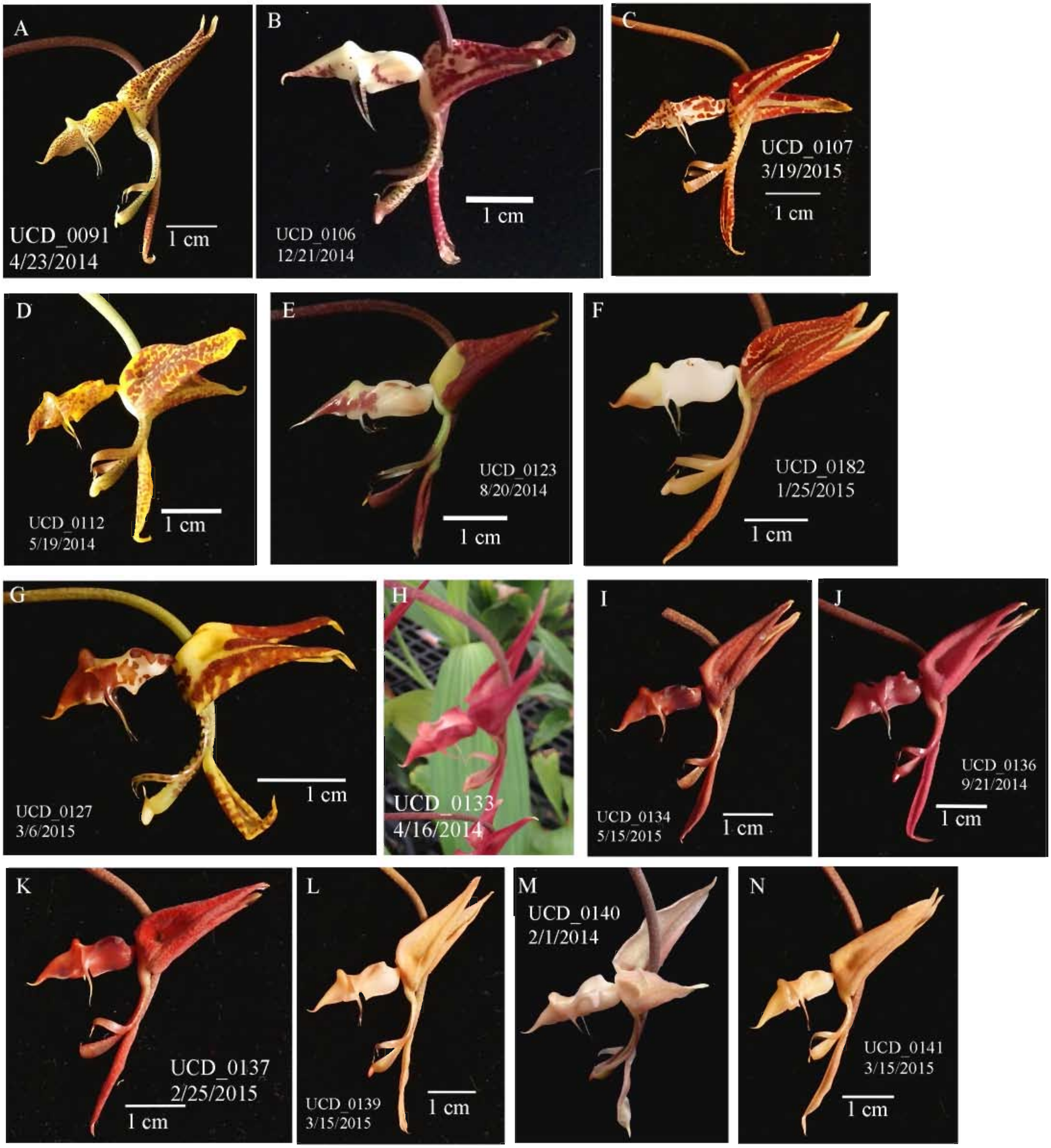

Figure S4: *Gongora* species belonging to subgenus *Gongora* section *Gongora* continued. **(A)** *G. nigrita*. **(B,C)** *Gongora* aff. *quinquernervis*. **(D)** *G. rufescens* **(E-G)** *Gongora superflua*. **(G)** *Gongora tricolor*. **(H-N)** *Gongora* aff. *unicolor* (UCD_0135 not pictured).

Section *Gongora* continued

Gongora pleiochroma-- beta bisabolene chemotype

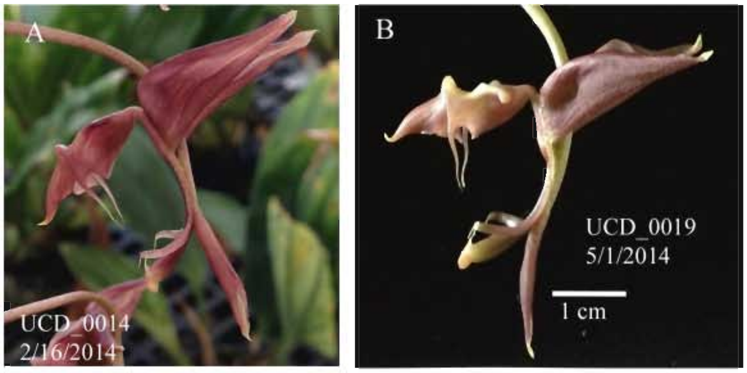

Gongora pleiochroma-- linalool chemotype

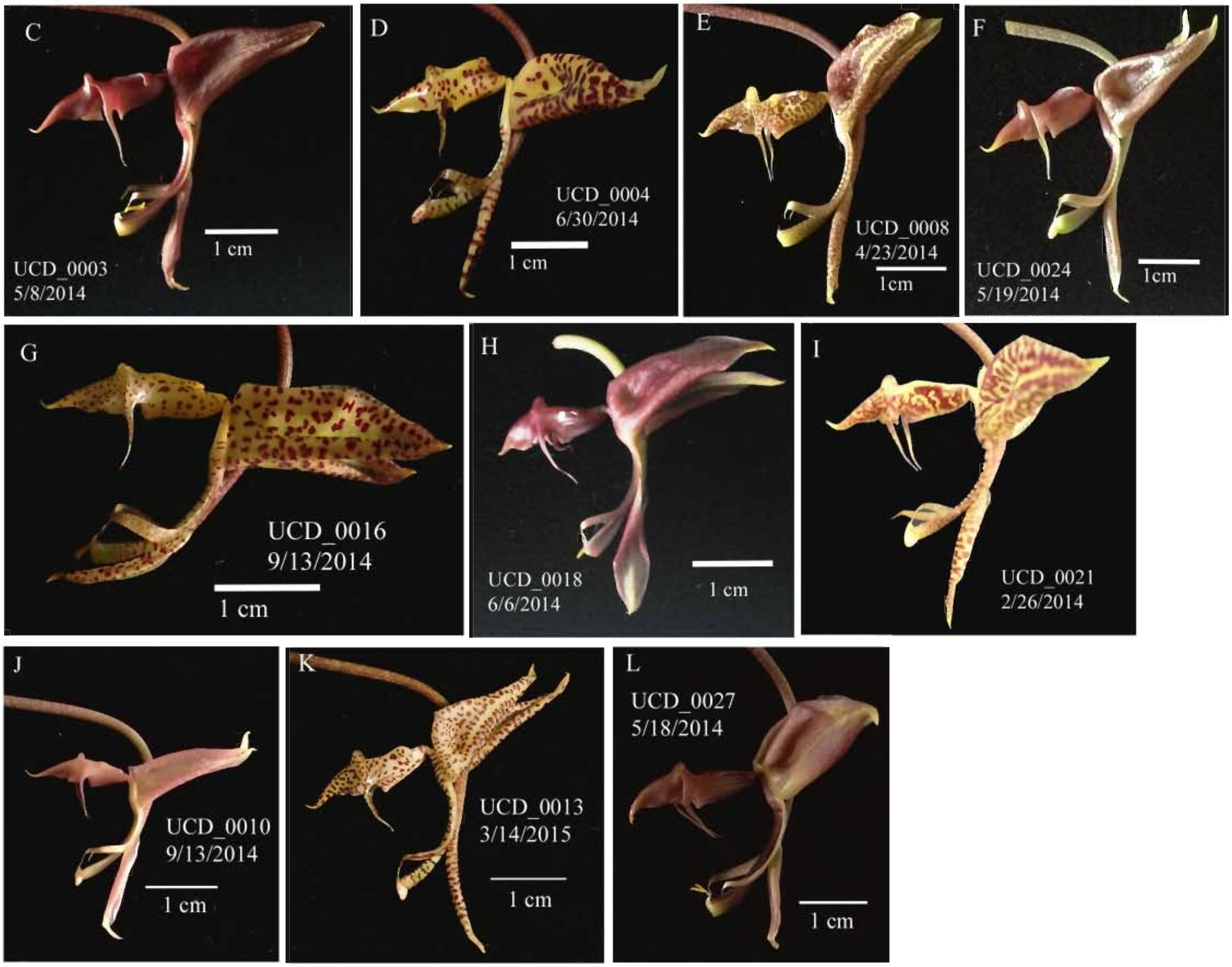

Figure S5: *Gongora* species belonging to subgenus *Gongora* section *Gongora* continued. *Gongora pleiochroma* chemotypes. **(A,B)** *Gongora pleiochroma* with chemotype of beta bisabolene. **(C-L)** *Gongora pleiochroma* with linalool chemotype.

Section *Gongora* continued

Gongora pleiochroma-- beta ocimene chemotype

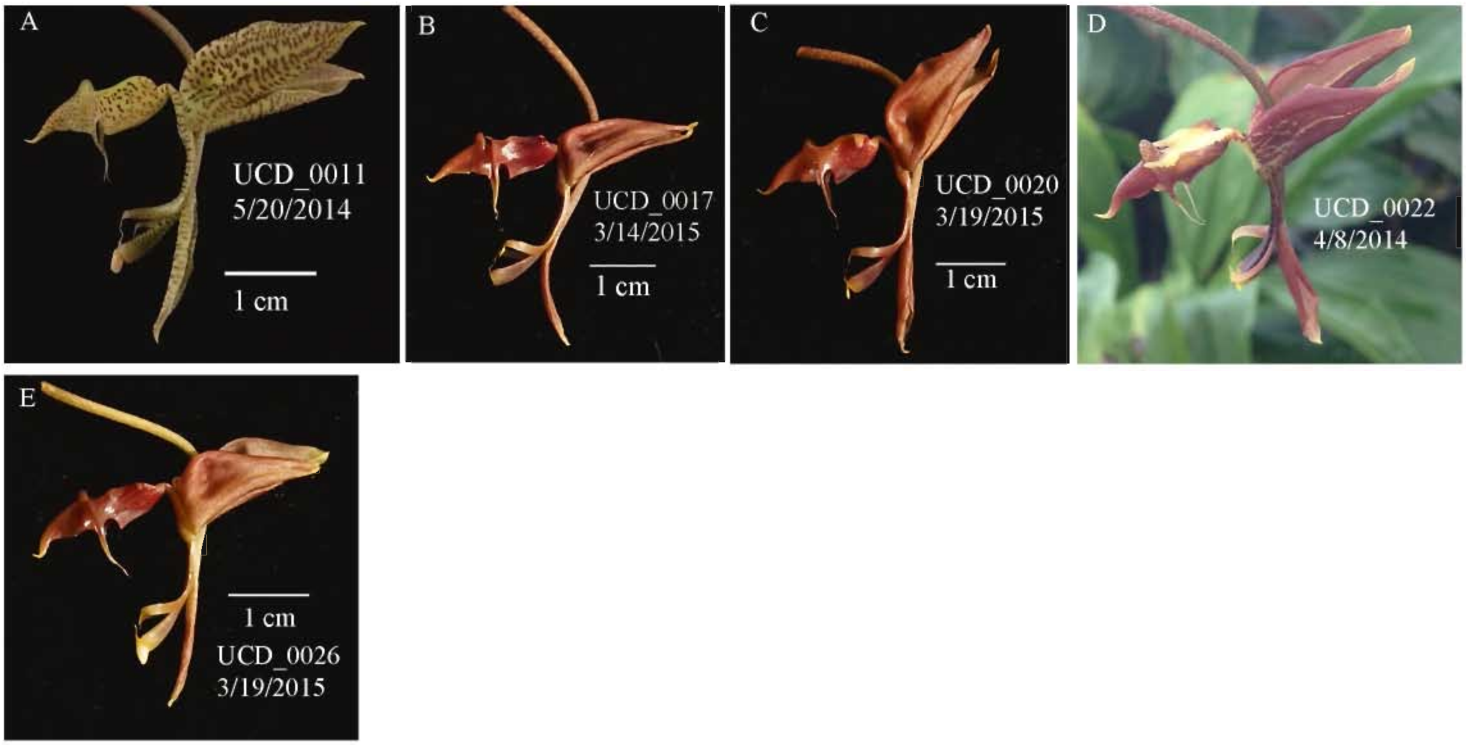

Gongora pleiochroma -- unidentifiable chemotype

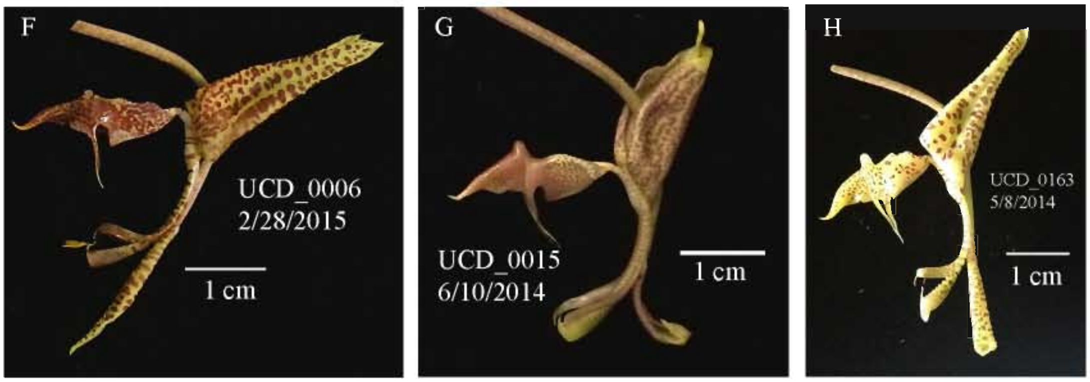

Figure S6: *Gongora* species belonging to subgenus *Gongora* section *Gongora* continued. *Gongora pleiochroma* chemotypes. **(A-E)** *Gongora pleiochroma* with chemotype of beta ocimene. **(F-H)** *Gongora pleiochroma* with unidentifiable chemotype.

Section *Gongora* continued

Gongora species chemotype A.

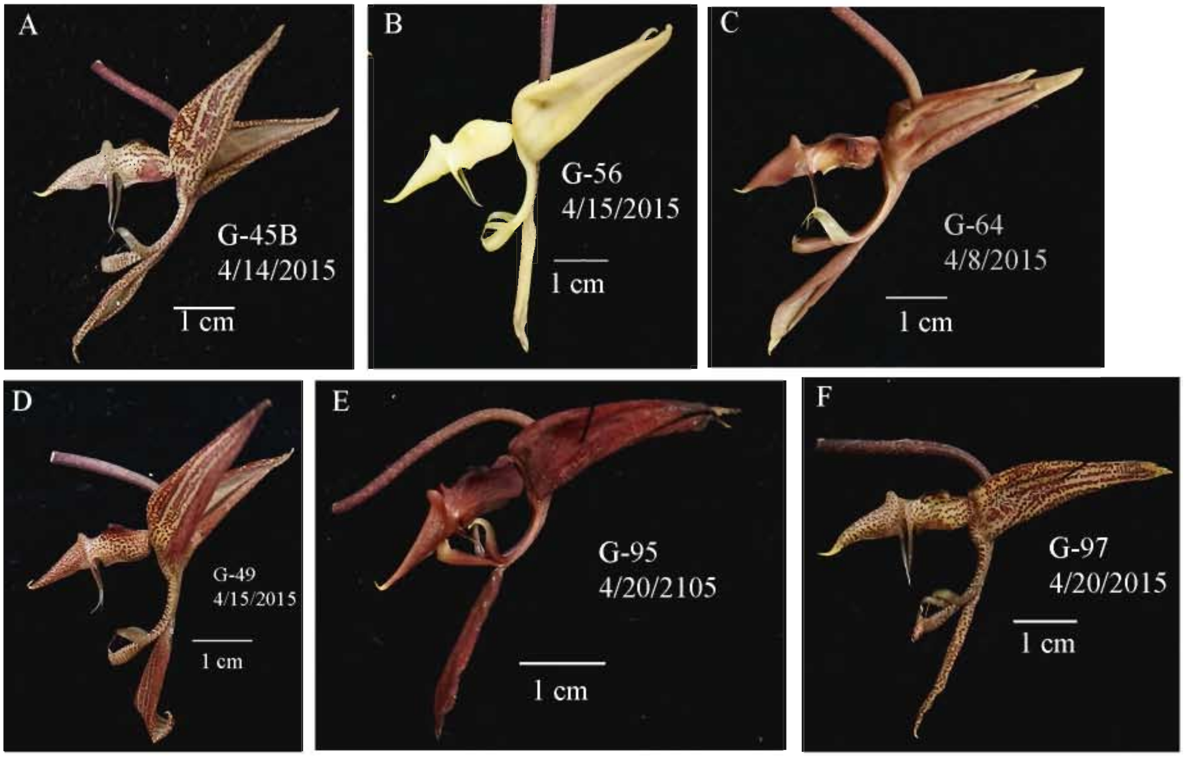

Gnngora species chemotype M

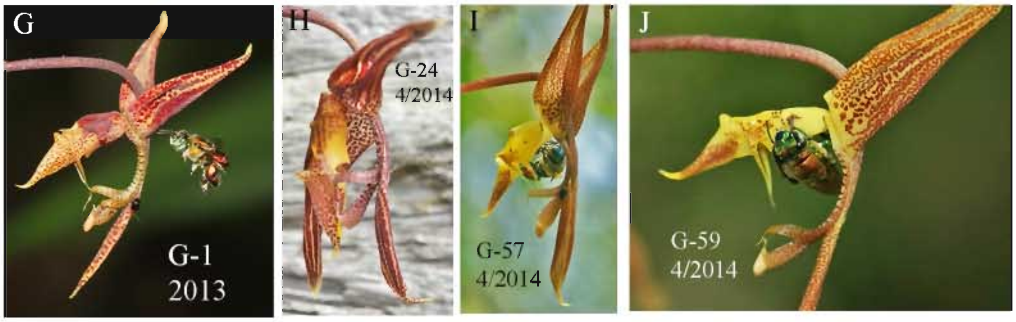

Gongora species chemotype S

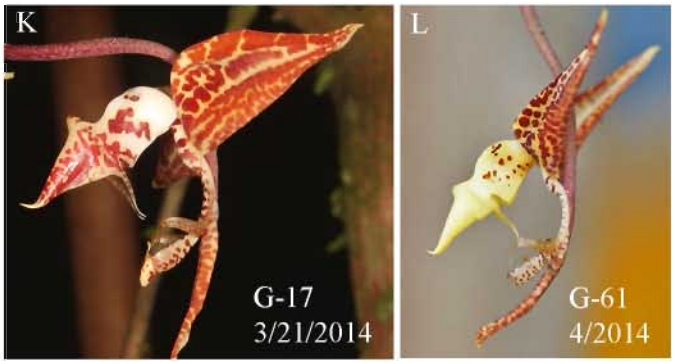

Figure S7: *Gongora* species belonging to subgenus *Gongora* section *Gongora* continued. *Gongora* speciescollected in sympatry from the Esquinas Rainforest near Golfito located on the southern Pacifiic coast of Costa Rica.
(A-F). *Gongora* sp. chemoptype A. **(G-J)** *Gonogra* sp. chemotype M. **(K,L)** *Gongora* sp. chemotype S .

Section *Grossa*

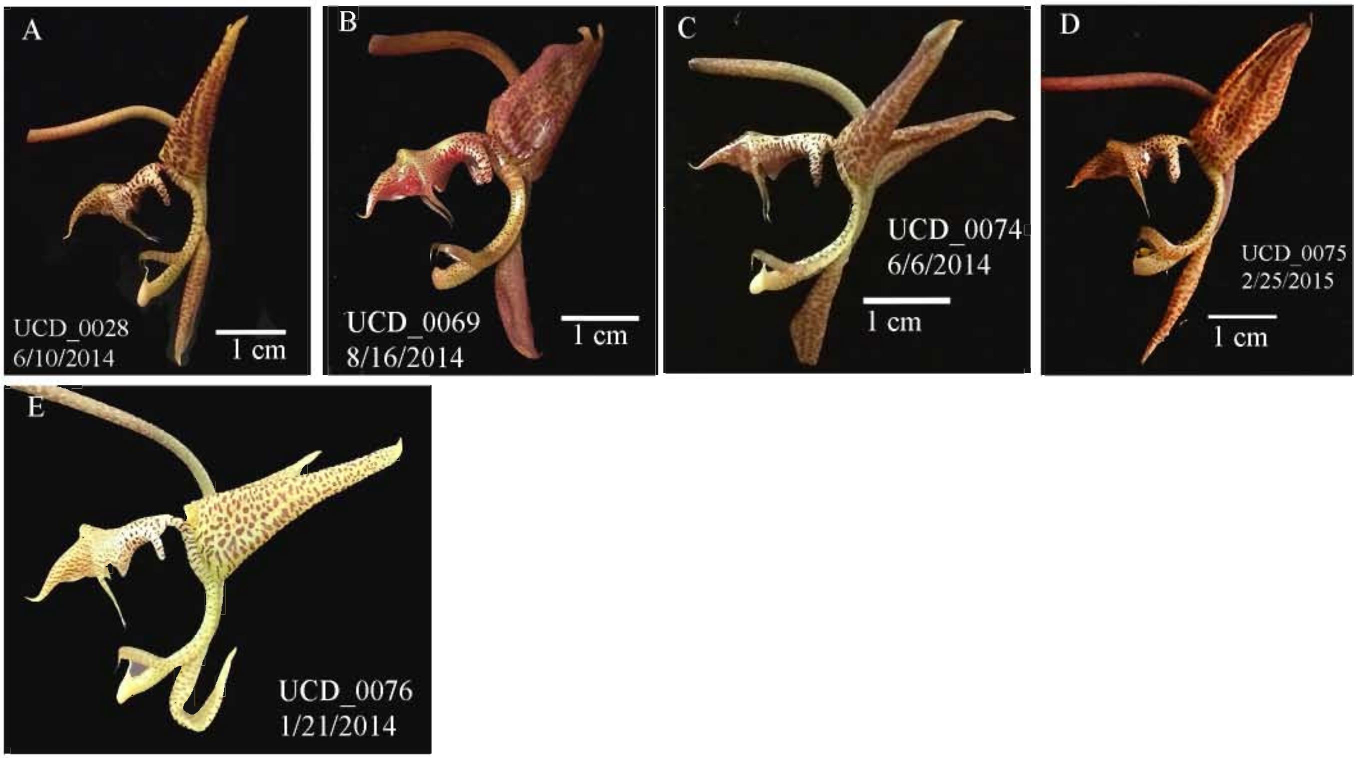

Section *Truncata*

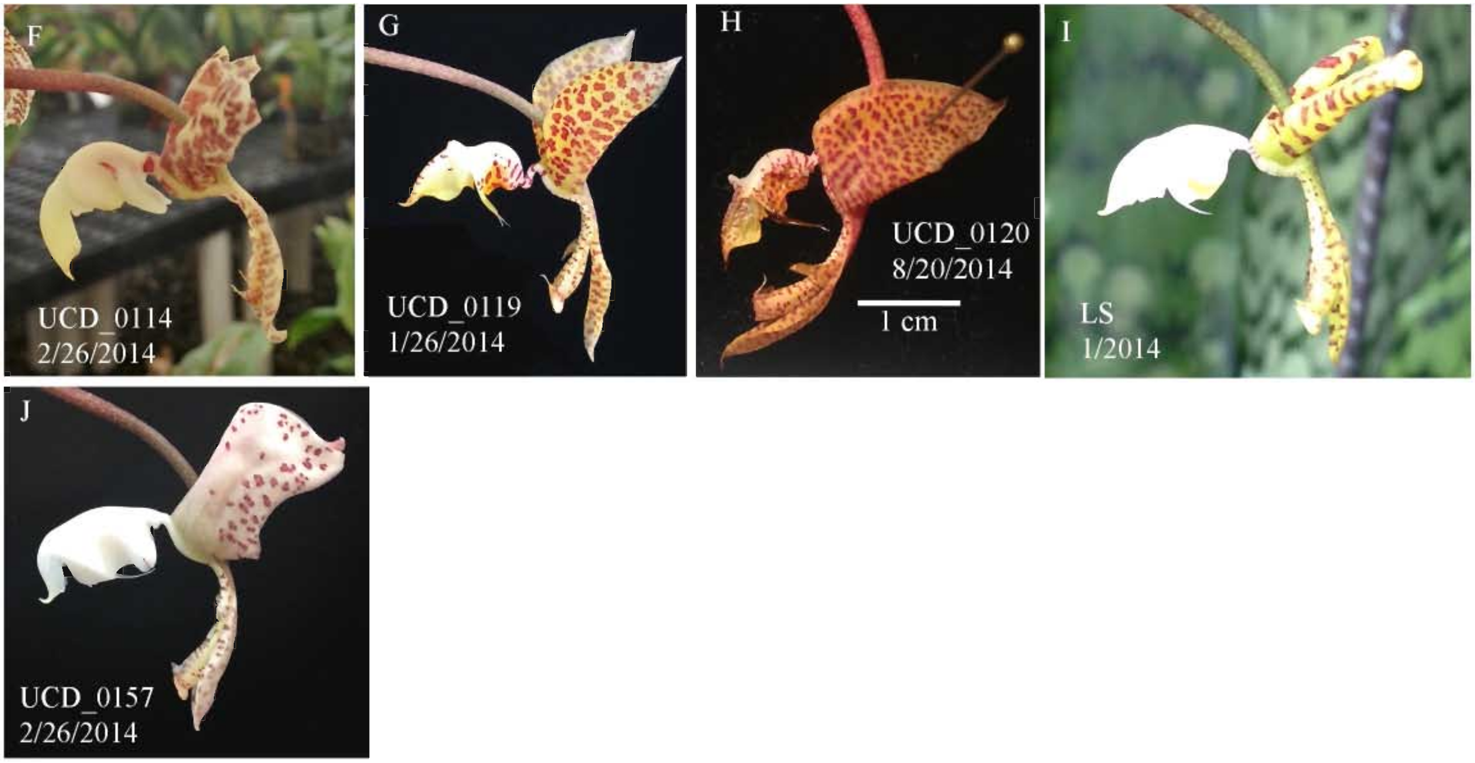

Figure S8: *Gongora* speices belonging to subgenus *Gongora* sections *Grossa* and *Truncata.* (A-E) *Gongora grossa.* (F) *Gongora scaphephorus.* (G-H) *Gongora sphaerica.* (I) *Gongora tracyana.* (J) *Gongora truncata.*
