# Supplementary material for: Evolution and diversity of floral scent chemistry in the euglossine bee-pollinated orchid genus Gongora
Source: Ann Bot. 2016 May 30;118(1):135–48. doi: 10.1093/aob/mcw072 (PMC4934395; doi:10.1093/aob/mcw072)

Cluster Dendrogram of Replicates
chemical dist=Bray-Curtis; clustering method=UPGMA
format: Species/Individual/Sample Number (MH_XXX)/Day of flowering

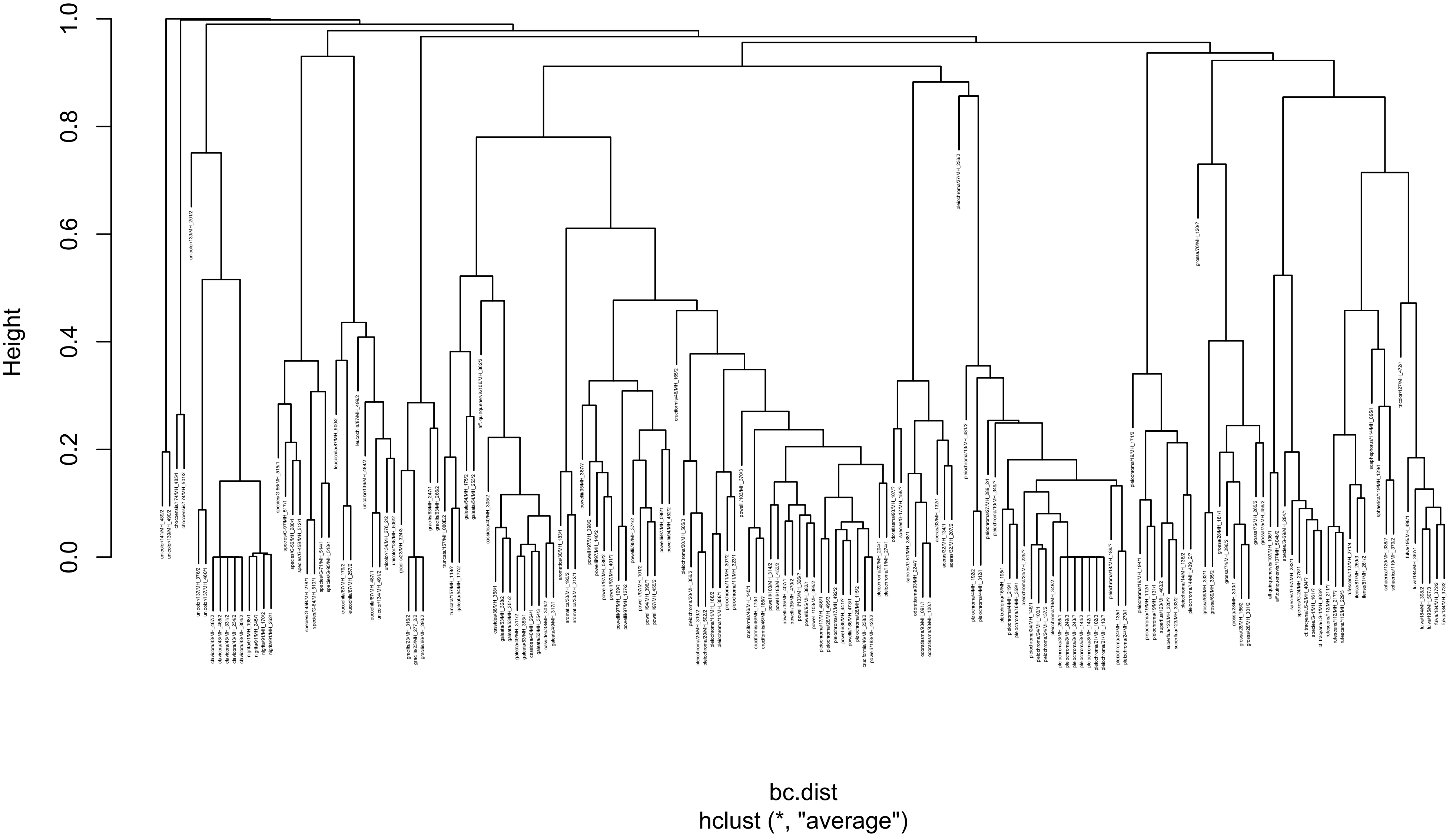

Supplement: Supplementary Data [file supp_mcw072_suppl_data.zip › aob-15945-s02.pdf]
